# Post-translational modifications of immune checkpoints: unlocking new potentials in cancer immunotherapy

**DOI:** 10.1186/s40164-025-00627-6

**Published:** 2025-03-14

**Authors:** Qiongjie Hu, Yueli Shi, Huang Wang, Liuwen Bing, Zhiyong Xu

**Affiliations:** 1 Department of Respiratory and Critical Care Medicine, The Fourth Affiliated Hospital of Zhejiang University School of Medicine, Yiwu, 322000 Zhejiang Province China; 2 Zhejiang Key Laboratory of Precision Diagnosis and Treatment for Lung Cancer, Yiwu, 322000 China; 3 The Third Affiliated Hospital of Zhejiang, Chinese Meical University, Hangzhou, 310013 China; 4 Department of Respiratory & Critical Care Medicine, The First Affiliated Hospital of Nanjing Medical University, Nanjing, China

**Keywords:** Cancer immunotherapy, Immune checkpoint blockade, Post-translational modifications, Ubiquitination, Phosphorylation

## Abstract

Immunotherapy targeting immune checkpoints has gained traction across various cancer types in clinical settings due to its notable advantages. Despite this, the overall response rates among patients remain modest, alongside issues of drug resistance and adverse effects. Hence, there is a pressing need to enhance immune checkpoint blockade (ICB) therapies. Post-translational modifications (PTMs) are crucial for protein functionality. Recent research emphasizes their pivotal role in immune checkpoint regulation, directly impacting the expression and function of these key proteins. This review delves into the influence of significant PTMs—ubiquitination, phosphorylation, and glycosylation—on immune checkpoint signaling. By targeting these modifications, novel immunotherapeutic strategies have emerged, paving the way for advancements in optimizing immune checkpoint blockade therapies in the future.

## Introduction

Tumor microenvironment (TME) refers to the local environment in which tumor generation and development occur. The TME has complex components, including tumor cells, blood vessels, immune cells, fibroblasts, various signaling molecules, etc. [1]. These components interact to promote tumor growth while increasing tumor heterogeneity, adaptability and metastasis. Recognized for its role in tumor progression, current research focuses on remodeling the TME to combat cancer effectively, particularly through immunotherapeutic approaches targeting the microenvironment to restore immune cell anti-tumor functions [2].

**Fig. 1:**
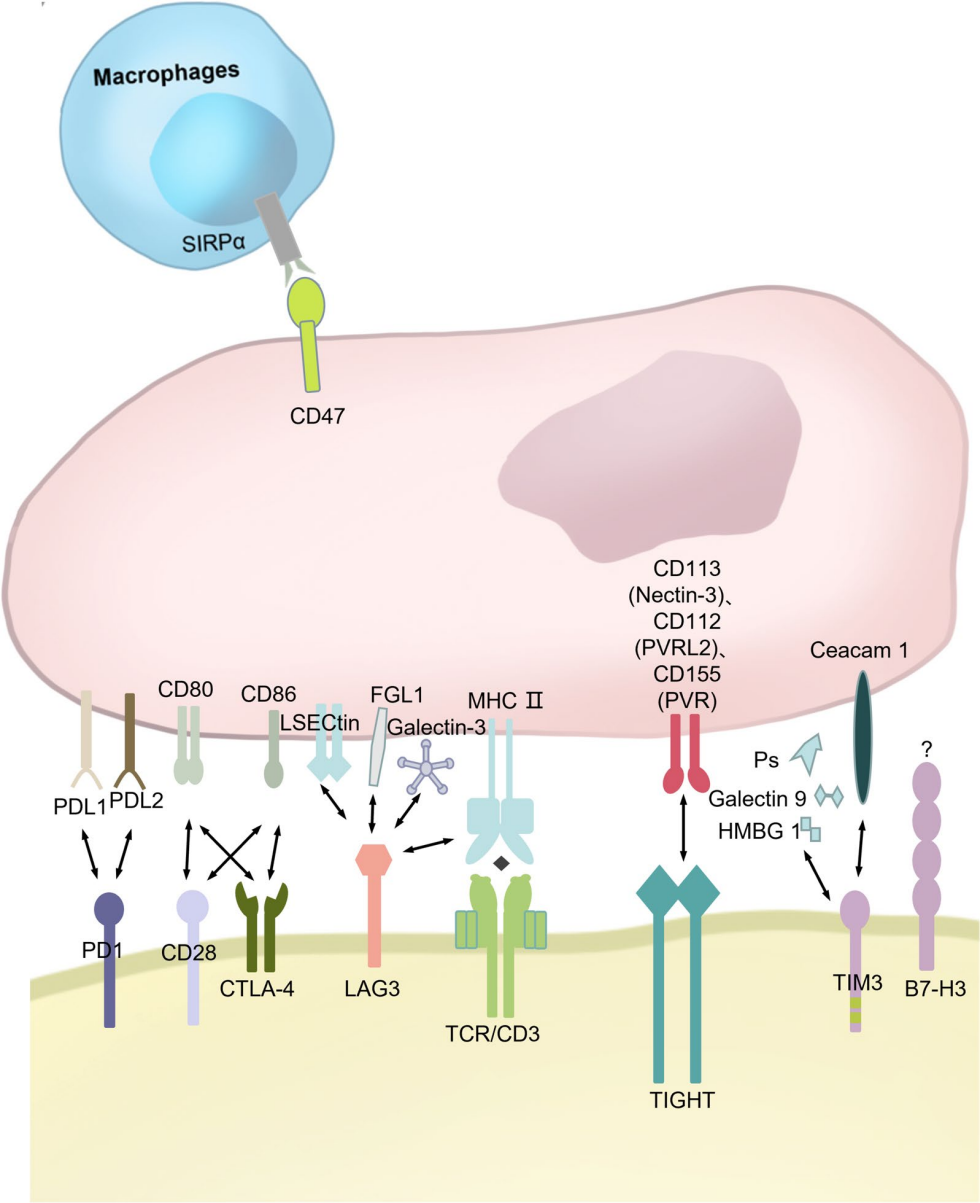
Checkpoints and their ligands: Diverse ligand-receptor interactions, including immune checkpoints and their associated ligands. *PD-1* Programmed Cell Death protein 1, *PD-L1* Programmed Death Ligand 1, *PD-L2* Programmed Death Ligand 2, *LAG-3* Lymphocyte Activation Gene 3, *CD28* Cluster of Differentiation 28, *CD47* Cluster of Differentiation 47, *CD80* Cluster of Differentiation 80, *CD86* Cluster of Differentiation 86, *CD112 (Nectin-3)* Cluster of Differentiation 112, *CD113 (PVRL2)* Cluster of Differentiation 113, *CD155 (PVR)* Cluster of Differentiation 155, *LAG3* Lymphocyte-activation gene 3, *LSECtin* Liver sinusoidal endothelial cell lectin, *FGL1* fibrinogen-like protein 1, *MHC* Major histocompatibility complex, *TCR* T cell receptor, *B7-H3 (CD276)* immunoregulatory protein B7-homologue 3, *TIM-3 (HAVCR2)* T cell immunoglobulin and mucin domain-containing molecule 3, *TIGIT* T cell immunoreceptor with Ig and ITIM domains, *CTLA-4* cytotoxic T-lymphocyte-associated protein 4, *Ps* Phosphatidylserine, *HMGB 1* High mobility group box 1 protein

Tumor immunotherapy is grounded in the principle that, under physiological conditions, immune cells possess the ability to recognize and eliminate tumor cells. Nevertheless, tumors and their associated microenvironments generate a substantial quantity of immunosuppressive regulatory molecules. These molecules impede the functionality of immune cells and thereby facilitate the process of tumor immune escape [3]. To counter this, reactivating the host's immune system to recognize and eliminate tumor cells is critical. The upregulation of immunosuppressive ligand expression is now believed to be one of the primary causes of immunosuppression. This class of ligands mediates tumor immune escape by binding to receptors on the surface of immune cells and impeding immune cell function. For example, numerous studies have shown that tumor cells bind to the PD-1 receptor on the surface of T cells through high expression of PD-L1, resulting in suppression of the anti-tumor activity of T cells [4]. Such receptors and ligands are called "immune checkpoints". A large number of immune checkpoints have been identified and studied, such as PD-L1, PD-1, PD-L2, LAG-3, CD47, TIM-3, TIGIT, B7-H3, CTLA-4, etc. [5] (Fig. 1). Given that immune checkpoints are surface—expressed molecules, their interactions can be efficiently blocked by the corresponding antibodies. Such a property has paved the way for the development of immune checkpoint blockade (ICB) therapies, which have emerged as a crucial therapeutic strategy in the field of cancer immunotherapy. This therapy is one of the hottest immunotherapies available and focuses on restoring the anti-tumor capacity of immune cells by blocking the binding of immunosuppressive receptors and ligands. The most effective and typical ICB therapy is anti-PD-1/PD-L1 therapy, which has been approved by the FDA for the treatment of melanoma, lung cancer, liver cancer, gastric cancer, kidney cancer and many other tumors.

Despite the widespread use of immune checkpoint blockade (ICB) therapy, the effective response rate of patients is extremely low, only about 10–30%. In colorectal cancer patients, the response rate is even lower than 5% [6]. Meanwhile, systemic immunosuppressive blockade causes severe side effects to patients, involving various organs such as the gastrointestinal tract, skin, and liver [7]. In addition, there is still a lack of accurate predictive biomarkers of immunotherapy efficacy, making it impossible to effectively screen the population for immunotherapy benefit. In-depth investigation of the regulatory mechanisms of immune checkpoints is important for finding new biomarkers, developing new combination therapy strategies and improving the efficacy of current ICB therapy [8, 9].

Post-translational modifications (PTMs) of proteins refer to the process of covalent addition of functional groups or proteins to proteins, which in turn regulates the diversity, stability or localization of protein functions. The current mainstream PTMs include phosphorylation, ubiquitination, acetylation, glycosylation, SUMOylation, palmitoylation etc. [10]. In recent years, numerous studies have revealed that protein PTMs can affect their protein synthesis and membrane stability, the ability to bind ligands or monoclonal antibodies, as well as the effects on downstream signaling activation, ultimately leading to tumor immune escape and immune checkpoint inhibitor treatment resistance [11, 12]. For example, it has been shown that N-glycosylation of PD-L1 stabilizes PD-L1 and thus allows it to escape degradation by the proteasome [13, 14]. The PTMs of immune checkpoints are precisely regulated by multiple signaling pathways including the ubiquitination/deubiquitination signaling pathway [15], the phosphorylation/dephosphorylation signaling pathway, and the glycosylation/deglycosylation signaling pathway, among others. This paper presents a review of the known PTMs of major immune checkpoints and explores the potential for clinical translation with the aim of providing new ideas for anti-tumor immunotherapy.

## Major types of protein PTMs

### Definition of PTMs

**Fig. 2:**
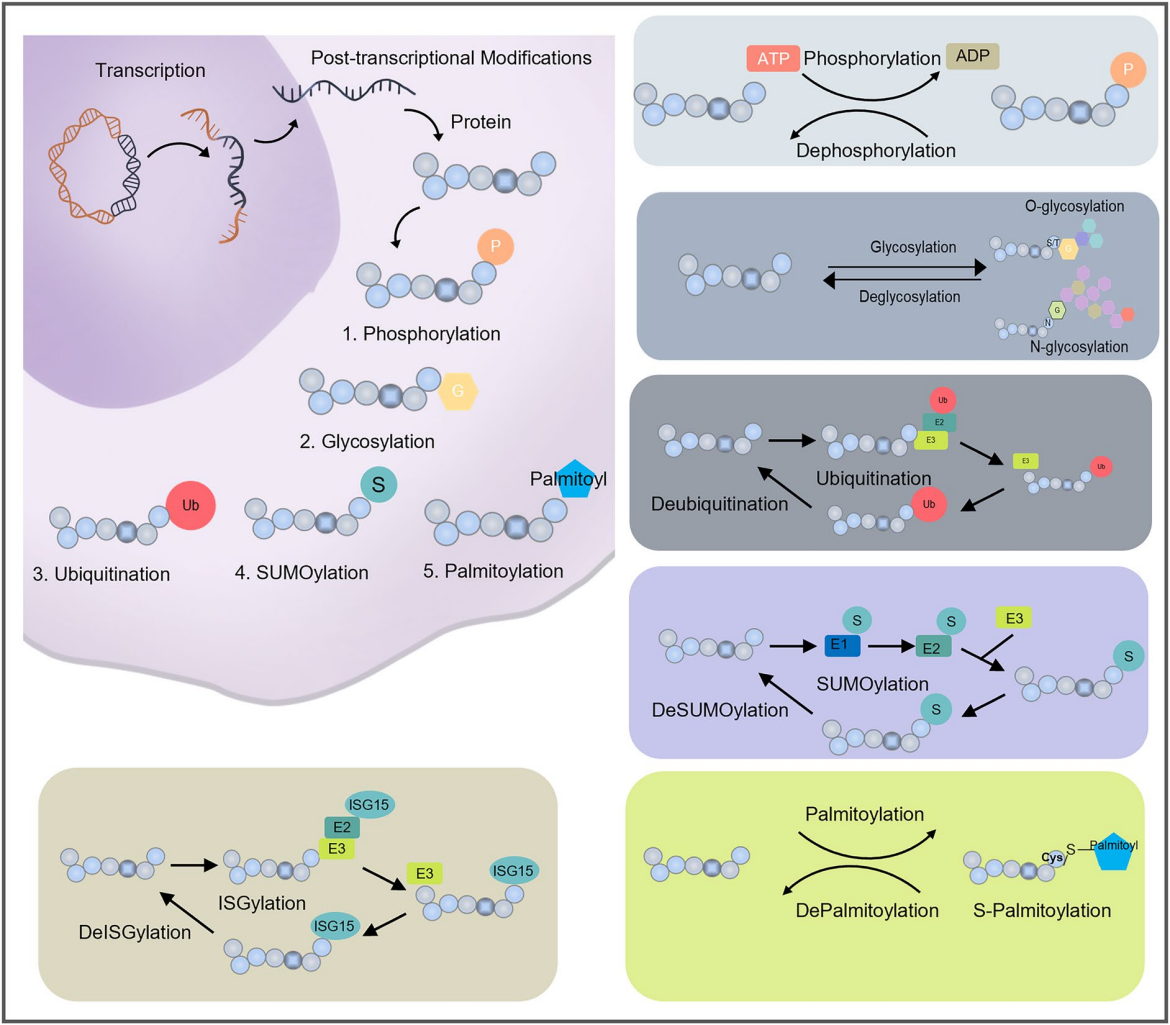
PTMs and their common types, including phosphorylation, glycosylation, ubiquitination, SUMOylation, palmitoylation and ISGylation: After DNA is transcribed into RNA, it is then translated into proteins and then PTM occurs. Currently, the main PTMs include phosphorylation modification, glycosylation modification, ubiquitination modification, SUMOylation, ISGylation and palmitoylation. Glycosylation (G, yellow hexagons), phosphorylation (P, orange circles), ubiquitination (Ub, red circles), SUMOylation (S, light blue circles), palmitoylation (palmitoyl, dark blue pentagon), ISGylation (ISG15, blue oval). *ATP* Adenosine TriPhosphate, *ADP* Adenosine DiPhosphate

PTM, also known as covalent modification, refers to the process of chemical modification of proteins after translation and plays an important role in almost all cellular signaling pathways and networks. In addition to enabling human gene coding diversity through specific mRNA splicing, PTM of proteins on the side chain or backbone promotes increased complexity from the genomic level to the proteomic level, which is key to proteomic diversity [16–19]. PTMs can be reversible and dynamic, and they have the ability to alter protein function. PTMs can alter protein function by proteolytic cleavage, adding various functional groups to amino acids, or altering the chemical properties of amino acid residues, thereby modulating protein abundance and/or activity and affecting protein interaction and downstream signaling pathways. There are hundreds of known ways to covalently modify proteins. The most common types of PTMs are phosphorylation, acetylation, glycosylation, ubiquitination, SUMOylation, palmitoylation, etc. [20, 21] (Fig. 2). Different PTMs play distinct roles in various biological processes. For example, reversible phosphorylation underlies many signal transduction pathways, glycosylation regulates the stability and structure of many membranes and secretory proteins, and ubiquitination targets proteins for degradation. These processes are performed and fine-tuned by thousands of enzymes, and when these enzymes become dysregulated, they can lead to pathology. In particular, in cancer cells, almost all major drivers from oncogenes and tumor suppressors to transcription factors and signaling molecules are associated with PTM dysregulation [22–24].

### Phosphorylation

Protein phosphorylation is one of the most widespread and important PTMs, which has been well-studied over the past few decades. Protein phosphorylation is reversible during signal transduction. Approximately 40% to 60% of the protein is temporarily phosphorylated. And thousands of different phosphorylation sites have been identified [25]. The balance between activation and inactivation of many key signaling molecules are delicately maintained by phosphorylation, and dysregulation of these processes can lead to disruptions in signal transduction and metabolism [26]. Phosphorylation is the process of transferring the phosphate group of ATP to the amino acid residues of the substrate protein (serine, threonine, tyrosine), catalyzed by protein kinase [27]. The reverse process, on the other hand, involves the removal of the corresponding phosphate group by protein phosphatases. It is the opposite action of these two enzymes and the energy expenditure and generation involved in them that make phosphorylation the preferred mode of regulation of many physiological activities in the body (Fig. 2). Complex enzyme phosphorylation networks play a key role in cellular cascade reactions. Phosphorylation-regulated cell signaling affects almost all aspects of cellular processes, from growth, differentiation and apoptosis, etc. [28].

### Ubiquitination

Ubiquitination is the covalent linkage of ubiquitin (a highly conserved primary sequence linked to the lysine of the substrate protein by an isopeptide bond). Covalent modification of substrates through a three-step enzymatic cascade reaction: Ubiquitin is activated by ubiquitin-activating enzyme (E1) through the use of ATP. The activated ubiquitin is then transferred to ubiquitin-conjugating enzyme (E2) and recruited by E3 ligase, which binds the substrate protein to ubiquitin molecules and ultimately targets the substrate protein to the proteasome for endogenous degradation- the system is known as the ubiquitin–proteasome system (UPS) (Fig. 2). Its precise regulation ensures the removal of unwanted, damaged, misfolded and potentially harmful proteins [29, 30]. Depending on the number of ubiquitin units attached, proteins can be monoubiquitinated (addition of a single ubiquitin molecule) or polyubiquitinated (sequential addition of more ubiquitin molecules to the previous one). Usually, ubiquitination can degrade substrates through the K48-linked chain, while the K63-linked chain is mainly involved in non-proteasome degradation functions. In contrast, the assembly of ubiquitin on the substrate can be reversed by the action of Deubiquitinases (DUBs), which dissociate ubiquitin from the ubiquitinated target proteins. The process of deubiquitination is very precise and well-ordered and is essential for maintaining physiological ubiquitin homeostasis. It is involved in a variety of important life activities including cell cycle regulation, DNA repair, gene transcription, protein degradation and kinase activation.

### Glycosylation

Glycosylation, a highly diverse PTM, plays a crucial role in enhancing protein diversity. This process, involving hundreds of enzymes and a vast array of glycans, significantly increases protein structural complexity and is essential in various biological processes [31–34]. Glycosylation typically occurs in membrane and secreted proteins, with N-linked (addition of glycans to asparagine residues in the ER) and O-linked glycosylation (attachment of glycans to serine/threonine residues in the Golgi apparatus) being the most common forms. Deglycosylation, the removal of saccharides from glycoproteins, can reverse this process (Fig. 2). Both glycosylation and deglycosylation are vital for proper protein folding, localization, stabilization, and function, thereby regulating recognition, adhesion and cell killing functions of immune cells [31–33, 35–42].

### SUMOylation

SUMOylation is the process of adding SUMO to proteins. SUMOylation is also a reversible post-translational modification that can be reversed by specific proteases called SUMO-specific proteases (SENP), such as SENP1、SENP2、SENP 3. Currently, four SUMO isoforms have been identified, namely SUMO1, SUMO2/3, and SUMO4. The SUMO catalytic cycle comprises maturation, activation, conjugation, ligation, and de-modification. The dysregulation of the SUMO system is associated with various diseases, particularly cancer. SUMOylation is widely involved in carcinogenesis, DNA damage responses, cancer cell proliferation, metastasis, and apoptosis. Therefore, SUMO can serve as a potential therapeutic target for cancer. Some studies have found that IR + ATRi can activate the classical cGAS-STING-pTBK1/pIRF3 axis by increasing the level of cytoplasmic double-stranded DNA and activate non-classical STING signaling by attenuating the SHP1-mediated inhibition of the TRAF6-STING-p65 axis, and by promoting the SUMOylation of SHP1 at lysine 127 [43].

### Palmitoylation

Protein palmitoylation is a lipid modification that is usually catalyzed by members of the zinc finger aspartate-histidine-histidine-cysteine (ZDHHC) motif-containing palmitoyltransferase family. The main form is the S-palmitoylation modification, which involves the covalent addition of a 16-carbon fatty acid, palmitic acid to the thiol group of cysteine residues [44]. The attachment of the hydrophobic lipid group influences the membrane anchoring of the target protein and regulates its activity, interaction, trafficking, localization, and stability [44].

### ISGylation

ISGylation is a newly discovered ubiquitin-like post-translational modification mediated by the ISG15 protein encoded by interferon-stimulated genes (ISGs) and its specific enzyme system (E1-activating enzyme UBE1L, E2-binding enzyme UBCH8, E3-ligating enzyme HERC5, TRIM25 or ARIH1, etc.). ISGylation can inhibit protein degradation by competitively binding to the ubiquitination site [45]. ISGs encode all ISG15 conjugating and deconjugating enzymes. Their expression is regulated by interferons [46]. This regulatory mechanism by interferons endows ISGylation with an important function in tumor immunity.

PTMs increase the complexity from the genome to the proteome, either synergistically or exclusively modifying proteins. These modifications alter a protein's physicochemical properties, spatial conformation, stability, and function, thus regulating its biological activity. As key regulators in tumor immune escape, PTMs of immune checkpoints play a significant role in their maturation, degradation, and translocation. This summary highlights key PTMs and mechanisms involved in the regulation of classical immune checkpoints.

## Modifications of immune checkpoints and the underlying mechanisms

### PD-1/PD-L1, PD-L2

**Fig. 3:**
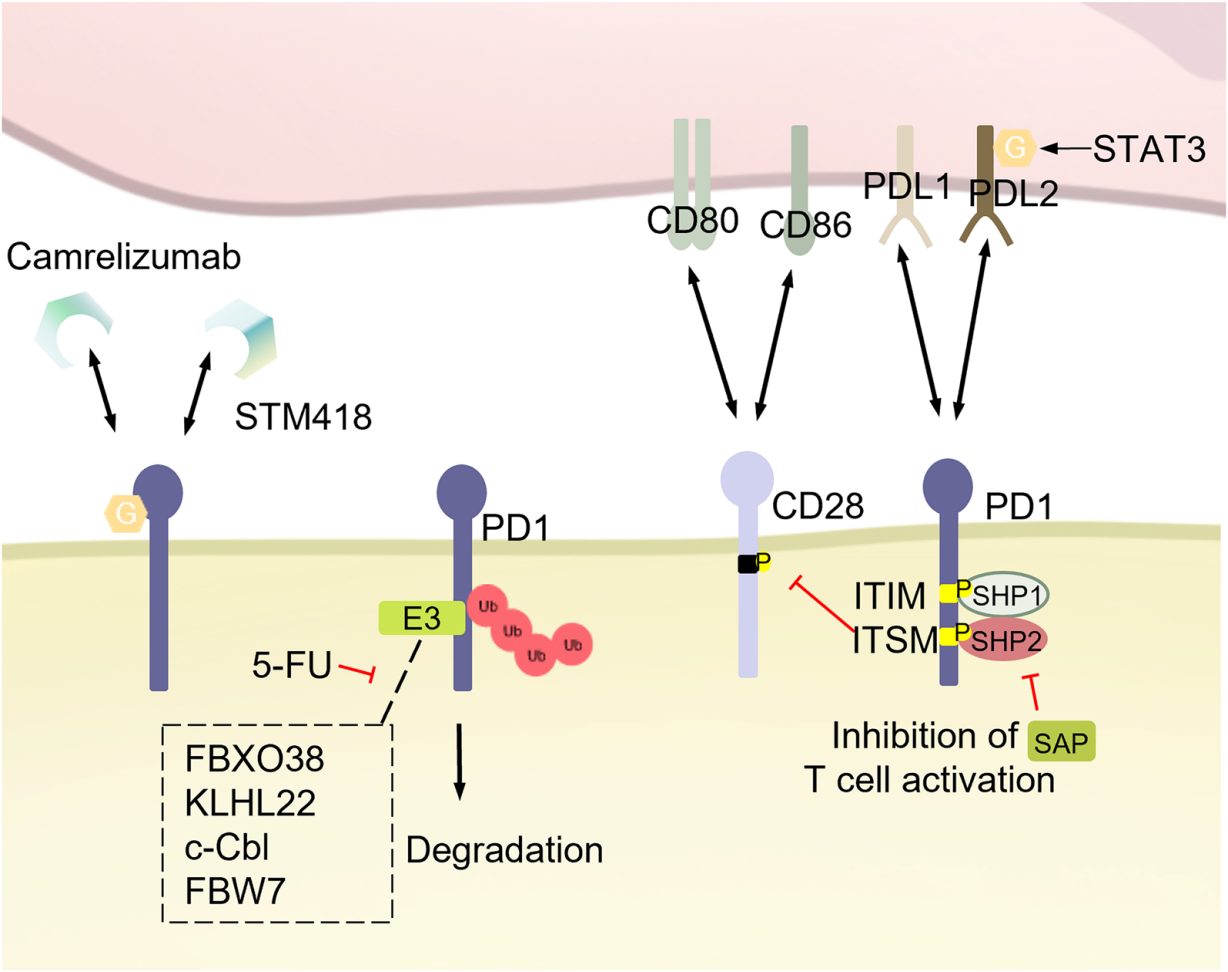
PTMs of PD-1: PD-L1 and PD-L2 are ligands of PD-1. PD-1 inhibits CD28 signaling by recruiting the protein tyrosine phosphatases SHP2/SHP1 through phosphorylated ITSM/ITIM. SAP suppressed PD-1 signaling by inhibiting the activity of SHP2. E3 ubiquitin ligases, such as FBXO38, KLHL22, c-Cbl and FBW7, promote the ubiquitination and degradation of PD-1 by interacting with it. 5-FU can block the ubiquitination process of PD-1. Camrelizumab and STM418 are antibodies that can target the N58 glycosylation site of PD-1. *PD-1* Programmed Cell Death protein 1, *PD-L1* Programmed Death Ligand 1, *PD-L2* Programmed Death Ligand 2, *CD28* Cluster of Differentiation 28, *CD80* Cluster of Differentiation 80, *CD86* Cluster of Differentiation 86, *SHP1* Src homology region 2 (SH-2) domain-containing phosphatase 1, *SHP2* Src homology region 2 (SH-2) domain-containing phosphatase 2, *STAT3* Signal Transducer and Activator of Transcription 3, *5-FU* 5-Fluorouracil, *FBXO38* F-box protein 38, *KLHL22* Kelch Like Family Member 22, *c -Cbl* Casitas B-lineage Lymphoma, *FBW7* F-box and WD repeat domain-containing 7

PD-1, an apoptosis-related gene discovered by T. Honjo and colleagues at Kyoto University in 1992 [47], is primarily expressed on immune cells as a co-inhibitory receptor. PD-1 plays a pivotal role in suppressing the immune response [48–50]. PD1, together with its ligands—PD-L1 (CD274, B7-H1) and PD-L2 (CD273, B7-DC), belongs to the B7-CD28 family. They are all type I transmembrane glycoproteins with immunoglobulin-like extracellular domains (Fig. 3). These molecules are involved in maintaining peripheral tolerance but can also hinder anti-tumor immunity [50–53]. By binding to PD-L1 and PD-L2, PD-1 inhibits T lymphocyte function, thereby suppressing autoimmune responses.

#### PD-1

As one of the most important immune checkpoints, PD-1 is a transmembrane protein expressed on activated T cells and its major ligands are PD-L1 and PD-L2 [54]. The binding of PD-1 to PD-L1 or PD-L2 inhibits T-cell activation, with PD-L2 having a 2–sixfold higher affinity for PD-1 than PD-L1, though PD-L1 is more broadly expressed [55]. Antibody therapies targeting PD-1 and its ligands are the most widely used immunotherapies in clinic today. Structurally, PD-1 comprises three main regions: the extracellular immunoglobulin variable (IgV) region, a hydrophobic transmembrane region, and an intracellular region that includes the immunoreceptor tyrosine-based inhibitory motif (ITIM) and the immunoreceptor tyrosine-based switch motif (ITSM) (Fig. 3) [56].

PD-1 contains two key tyrosine phosphorylation sites within the ITIM (Y223) and ITSM (Y248) regions. Upon ligand binding, these sites are phosphorylated by Src family kinases like Lck [57]. Phosphorylated PD-1 then recruits the phosphatase SHP2, which dephosphorylates critical molecules such as ZAP-70, CD3ζ, and PKCθ, leading to inhibition of TCR signaling and reduced T cell activation, thereby diminishing T cell anti-tumor activity [58, 59]. Recent research shows that in bone marrow cells, PD-1 can be induced by GM-CSF to phosphorylate, subsequently recruiting SHP2 to inhibit differentiation, activation, and anti-cancer functions [60]. In addition, PD-1 phosphorylation also regulates T cell function through PTEN-PI3K-AKT and RAS-MEK-ERK signaling [61, 62]. Building on this, Fernandes et al. developed a bispecific diabody (RIPR-PD-1) that induces the cis-linkage of PD-1 and CD45, reducing PD-1 phosphorylation, and showing greater effectiveness in tumor treatment than conventional anti-PD-1 therapies [63].

PD-1 is also a highly glycosylated protein, with four potential N-glycosylation sites identified on the extracellular IgV domain of human PD-1: N49, N58, N74, and N116. Mutating these sites to Glutamine (Q) significantly reduces the molecular weight of PD-1 [64]. The glycosylation level of PD-1 increases during T-cell activation [65], likely due to elevated expression of glycosyltransferase B3GNT2 and FUT8. Using CRISPR-Cas9 in T cells, Okada et al. found that core fucosylation modifications impact PD-1 surface expression, with all four glycosylation sites involved. Functional analysis indicates that glycosylation at N49 and N74 is essential for PD-1 surface expression and proper function. Mechanistic studies reveal that the core fucosyltransferase Fut8 drives this modification, and inhibiting Fut8 reduces PD-1 surface levels, thereby restoring T cell function [64]. In immunotherapy design targeting PD-1 glycosylation, Shi et al. developed a CAR-T with an N74 mutation, significantly enhancing CAR-T's anti-tumor activity by reducing PD-1 surface levels [66]. Additionally, the deletion of core fucosylation has been shown to promote ubiquitin-mediated degradation of PD-1, effectively eliminating T-cell suppressor signaling [37].

Glycosylation modifications also influence PD-1's ability to bind to ligands and specific monoclonal antibodies (mAbs). Studies have shown that the binding affinity of the FDA-approved PD-1-specific mAb Camrelizumab to PD-1 is affected by glycosylation, with strong binding observed to glycosylated PD-1. However, this binding is significantly reduced when the N58A mutation occurs [67, 68]. Additionally, mutations at glycosylation sites within the PD-1 IgV domain, particularly at N58, result in the loss of PD-1’s ability to bind to PD-L1, making N58 a critical site for regulating their affinity. Building on these findings, researchers developed an antibody targeting the PD-1 N58 glycosylation site, named STM418, which demonstrated significantly higher affinity for PD-1 than the existing mAb nivolumab [65].

FBXO38 is the first identified PD-1-specific E3 ubiquitin ligase, shown to mediate polyubiquitination at the PD-1 K233 site, leading to its proteasomal degradation. However, chronic TCR signaling within the TME downregulates FBXO38 expression, facilitating rapid tumor growth. IL-2 can restore FBXO38 levels, which subsequently reduces PD-1 expression and inhibits tumor growth [69]. Further studies have identified other E3 ligases KLHL22, c-Cbl, and FBW7 that promote PD-1 ubiquitination and degradation by interacting with PD-1 [70–72]. Mice deficient in c-Cbl exhibit increased PD-1 expression, leading to a more rapid growth of the tumor. Similarly, high FBW7 expression favors the development of "hot tumors". Conversely, 5-fluorouracil (5-FU) treatment inhibits KLHL22-mediated downregulation of PD-1, suggesting that combining 5-FU with PD-1 mAbs could be an effective therapeutic strategy. A deeper understanding of PD-1 ubiquitination could open new avenues for tumor treatment.

##### PD-L1 phosphorylation

Phosphorylation is pivotal in regulating PD-L1. Glycogen synthase kinase 3 beta (GSK3β) phosphorylates PD-L1 at threonine 180 (T180) and serine 184 (S184), leading to its degradation via the 26S proteasome. This process is influenced by factors such as epidermal growth factor (EGF), tyrosine kinase inhibitors (TKIs), MET and olaparib, which modulate GSK3β activity [14, 73–75]. Similarly, glycogen synthase kinase 3 alpha (GSK3α) facilitates PD-L1 degradation by phosphorylating serine 279 (S279) and serine 283 (S283), promoting its interaction with E3 ubiquitin ligase ARIH1 [76, 77].

Metformin and D-mannose enhance PD-L1 phosphorylation at serine 195 (S195) through AMP-activated protein kinase (AMPK) activation, causing abnormal glycosylation, endoplasmic reticulum (ER) retention, and degradation [78]. Under energy deprivation, AMPK-induced phosphorylation at S283 disrupts PD-L1's interaction with CKLF-like MARVEL transmembrane domain containing 4 (CMTM4), leading to PD-L1 degradation [79]. NIMA-related kinase 2 (NEK2) stabilizes PD-L1 by phosphorylating threonine 194 (T194) and threonine 210 (T210), especially in pancreatic cancer, preventing degradation by the UPS [80, 81].

Additionally, tyrosine phosphorylation at tyrosine 112 (Y112), driven by Janus kinase 1 (JAK1) following interleukin-6 (IL-6) stimulation, recruits the N-glycosyltransferase STT3A to catalyze PD-L1 glycosylation and maintain PD-L1 stability [82]. Inhibitors of the IL-6/JAK1 pathway, such as ruxolitinib, can downregulate PD-L1 to improve T-cell-mediated tumor killing and immunotherapy efficacy [82].

##### PD-L1 ubiquitination

Over the past decade, substantial evidence has demonstrated that PD-1 and PD-L1 protein expression is primarily regulated through ubiquitin-mediated proteasomal degradation, an ATP-dependent and highly selective pathway known as the UPS [83–87]. The UPS operates as a dynamic, bidirectional system, where ubiquitin molecules are conjugated to substrate proteins via the ubiquitin ligase cascade (E1-E2-E3), leading to ubiquitination. Once a polyubiquitin chain forms on a target protein, it is recognized and degraded by the 26S proteasome. Conversely, DUBs counteract this process by cleaving Ub molecules from substrates, thereby preventing their degradation.

Multiple E3 ubiquitin ligases, including β-TrCP [14], SPOP [88], TRIM21 [89], STUB1 [90], TNFAIP3 [91], HRD1 [92], ARIH1 [76], NEDD4 [93, 94], RNF125 [95], and MARCH8 [96], have been identified as key promoters of PD-L1 degradation through ubiquitination. DUBs, categorized into six families based on structural and functional characteristics—Ubiquitin Carboxyl-Terminal Hydrolases (UCHs), ovarian tumor proteases (OTUs), ubiquitin-specific proteases (USPs), the Josephin domain family (MJD), the JAB1/MPN/Mov34 protease family (JAMM), and the recently discovered single-cell chemoattractant protein-inducible family—play a crucial role in PD-L1 regulation [97]. Notably, DUBs such as CSN5[86], OTUB1 [98, 99], USP5 [100], USP7 [101, 102], USP8 [103], USP9X [104, 105], USP21 [106], USP22 [107] can enhance PD-L1 expression by removing ubiquitin chains, thereby stabilizing the protein on the cell surface.

The phosphorylation regulation dependent on GSK3β is crucial for the recognition of E3 ubiquitin ligases. Non-glycosylated PD-L1 forms a complex with GSK3β and β-TrCP, promoting PD-L1 to be phosphorylated at the T180/S184 sites, which in turn triggers subsequent ubiquitination degradation. Stabilization of PD-L1 by inactivation of GSK3β or inhibiting β-TrCP enhances tumor-immunosuppressive function and gives an advantage for tumor cell survival in an in vivo mouse model [14, 108].

Cullin-RING E3 ubiquitin ligases (CRLs), the largest family of E3 ligases in eukaryotes, regulate various cellular processes, including PD-L1 ubiquitination. SPOP, a CRL3 adapter protein, forms a complex with Cullin 3 to modulate PD-L1 levels [88, 109–111]. TRIM21 and SPOP both can destabilize PD-L1 through ubiquitination that dependent on cyclin—dependent kinases. Specifically, CDK4 can strengthen the ubiquitination of PD-L1 mediated by SPOP, whereas CDK5 can suppress the ubiquitination of PD-L1 induced by TRIM21 [112].

STUB1 (CHIP), a co-chaperone of Hsp90/Hsc70, facilitates K48-linked polyubiquitination of PD-L1 [90, 113], but its potential collaboration with HIP1R remains unclear. Genome-wide CRISPR-Cas9 screens have identified regulators like CMTM6, which prolongs PD-L1's half-life by inhibiting STUB1-mediated ubiquitination [84, 87]. Caspase 8 induces PD-L1 ubiquitination degradation by upregulating A20 (TNFAIP3) expression [91], and 5,7,4'-trimethoxyflavone can stabilize HRD1 to also induce PD-L1's ubiquitination and promote its degradation [114].

Ubiquitin ligases RNF125 and NEDD4 also catalyze PD-L1 degradation [87, 93–95]. MARCH8, a newly identified E3 ligase, is implicated in PD-L1 degradation, though its mechanism remains unknown [96].

Mithramycin A (MIT) may increase PD-L1 expression by inhibiting its ubiquitination, although the exact mechanism is unclear [115]. PD-L1 also undergoes monoubiquitination, leading to lysosomal degradation, distinct from its polyubiquitination-driven proteasomal degradation [73, 116, 117]. Palmitoylation by DHHC3 inhibits monoubiquitination and prevents PD-L1 degradation via the ESCRT pathway, but the specific protease regulating this process is still unidentified.

CSN5, a COP9 signalosome subunit induced by nuclear factor κB p65, is critical for PD-L1 stabilization in cancer cells, aiding immune evasion. CSN5 not only negatively regulates CRLs, but also acts as a DUB, directly deubiquitinating PD-L1 [86, 118, 119]. Natural inhibitors like curcumin and berberine destabilize PD-L1 by inhibiting CSN5 activities, thereby reducing immunosuppression [86, 120].

USP22 regulates PD-L1 through two mechanisms [107]. It binds to PD-L1 and directly deubiquitinates it, thereby promoting its stability [107, 121]. Additionally, USP22 influences PD-L1 expression via the USP22-CSN5-PD-L1 axis by removing ubiquitin chains from CSN5, stabilizing CSN5, and subsequently regulating PD-L1 levels [107].

USP8 has been found to be able to impede PD-L1 degradation via the ubiquitin–proteasome pathway [103, 122]. However, another study shows that USP8 inhibitors antagonize K48-linked ubiquitination, enhancing TRAF6-mediated K63-linked ubiquitination, thereby increasing PD-L1 abundance [123].

TMUB1 stabilizes PD-L1 by direct binding and competes with the E3 ligase HUWE1 to interact with PD-L1, inhibiting its polyubiquitination at K281 in the ER [124]. OTUB1 deubiquitinates K48-linked chains on PD-L1, stabilizing its protein levels. Depletion of OTUB1 reduces PD-L1, decreases PD-1 binding on tumor cells, and heightens tumor cell susceptibility to PBMC-mediated cytotoxicity [98, 99].

In addition to the aforementioned enzymes, several newly identified DUBs, including USP5 [100], USP7 [101, 102], USP9X [104, 105], and USP21 [106] have been shown to stabilize PD-L1 expression through deubiquitination. While these DUBs play a role in maintaining PD-L1 levels, their specific mechanisms of action require further investigation. It is also essential to explore which DUBs are more physiologically relevant in regulating PD-L1 ubiquitination and whether this regulation is dependent on the cellular or tissue environment, varying across different cancer types.

##### PD-L1 N-glycosylation

N-linked glycosylation, the attachment of oligosaccharides to specific asparagine (N) residues in proteins, plays a crucial role in PD-L1 stability and its interaction with the PD-1 receptor. PD-L1 is glycosylated at four key residues (N192, N200, and N219), which helps maintain its stability by inhibiting GSK3β-β-TRCP-mediated polyubiquitination, thereby promoting immune evasion by inhibiting the function of T-cells [13, 14, 125, 126]. Studies have shown that inhibiting N-glycosylation, such as through the use of 2-deoxyglucose (2DG) or a combination of metformin and 2DG, can reduce PD-L1 expression on the surface of breast cancer cells. This inhibition of glycosylation leads to the deglycosylation of PD-L1, counteracting low glucose concentration-induced PD-L1 upregulation [127]. The N-glycosyltransferase STT3 promotes PD-L1 N-glycosylation, which is essential for PD-L1 upregulation during epithelial-mesenchymal transition (EMT), contributing to cancer cell immune evasion in preclinical models [128]. Additionally, β-1,3-N-acetylglucosaminyl transferase 3 (B3GNT3) catalyzes the poly-N-acetyllactosamine repeats on PD-L1, which are critical for PD-1 binding, particularly at residues N192 and N200 [13].The splice variant of FKBP5 (FKBP51s) promotes PD-L1 glycosylation and stability, although the precise mechanisms remain unclear [129]. Inhibiting FKBP51s with the selective inhibitor SAFit reduces PD-L1 levels [130]. The EGF signaling pathway has also been implicated in promoting PD-L1 glycosylation [14]. Furthermore, antibodies targeting PD-L1 glycosylation, such as gPD-L1, can block the PD-1/PD-L1 interaction, enhance PD-L1 internalization and degradation, and exhibit potent antitumor activity in triple-negative breast cancer models when used in antibody–drug conjugates (ADCs) [13, 65].

Metformin activates AMP-activated AMPK and phosphorylates the S195 site of PD-L1, resulting in abnormal N-linked glycosylation of PD-L1, causing it to be retained in the ER, followed by ER-associated degradation (ERAD), which reduces the stability and membrane localization of PD-L1 [78]. In a breast tumor model, it was found that metformin combined with anti-CTLA4 treatment leads to significant improvements in tumor burden, survival rate, and CTL activity [78]. Similar to metformin, it was also found in the 4T1 breast tumor model that the combination therapy of D-mannose and anti-PD-1 can significantly inhibit tumor growth and prolong the lifespan of tumor-bearing mice [131]. Another study found that resveratrol has dual effects on the PD-1/PD-L1 pathway. On the one hand, it can inhibit N-glycosylation of PD-L1, leading to the accumulation of abnormal N-linked glycosylated forms of PD-L1 in the ER. On the other hand, resveratrol predicted to bind to the intracellular domain of PD-L1 and induce its dimerization, thereby interfering with the PD-L1/PD-1 interaction [132].

GLT1D1, an enzyme that transfers glycans to proteins, can stabilize PD-L1 through N-linked glycosylation, thereby promoting immunosuppression and tumor growth. Studies have shown that GLT1D1 may be a novel therapeutic target for treating B-NHL [133].

Interestingly, a recent study indicates that O-linked N-acetylglucosamine (O-GlcNAcylation) can promote tumor immune evasion by inhibiting the lysosomal degradation of PD-L1 [134]. Additionally, a previously undescribed site undergoing O-linked glycosylation was also found in the stalk region of the PD-1 protein [135]. Therefore, further research on the O-linked glycosylation of PD-L1/PD-1 may become a new direction for clinical diagnosis and treatment.

##### PD-L1 palmitoylation

Palmitoylation inhibits PD-L1 ubiquitination, thereby blocking its transport to the multivesicular body (MVB) via the ESCRT pathway, resulting in decreased lysosomal degradation and increased cell surface expression of PD-L1 and thus inhibiting the cytotoxicity of T cells. Existing studies have shown that the Cys272 site has been verified as a critical palmitoylation site of PD-L1, which contributes to the stability of PD-L1 and blocks the immune surveillance of T cells [44, 117, 136]. Studies indicate that ZDHHC3 and ZDHHC9 induce the palmitoylation of PD-L1 and stabilize its protein activity, thereby promoting tumor growth [117, 137]. Canceling palmitoylation by knocking out ZDHHC3/9 or introducing the C272A mutation in PD-L1 can reduce the expression and cell surface distribution of PD-L1 and make cancer cells sensitive to T cell-mediated killing in vitro [117, 138]. Additionally, researchers have also observed PD-L1 palmitoylation in cisplatin-resistant bladder cancer cells [136]. Inhibiting fatty acid synthase (FASN) can inhibit PD-L1 palmitoylation and its expression [136]. Therefore, targeting PD-L1 palmitoylation provides a new research direction for tumor therapy. Previous studies have demonstrated that targeting PD-L1 palmitoylation can increase the sensitivity of tumor cells to T cell killing and delay tumor growth [138, 139].

##### PD-L1 ISGylation

In LUAD, Qu et al. found that ISG15 expression positively correlated with lymphocyte infiltration, including CD3 + , CD4 + T cells, but not with CD8 + T cells. Mechanistic studies revealed that K-48-modified ISGylation was formed between ISG15 and PD-L1, thus increasing ubiquitination and protein degradation of PD-L1, activating anti-tumor immune function and sensitizing ICB efficacy [140].

##### Association of PD-L1 PTMs

PD-L1 activity is regulated by both glycosylation and ubiquitination, which are crucial for its stability and interaction with the PD-1 receptor. N-glycosylation at specific asparagine residues (N192, N200, and N219) is essential for maintaining PD-L1 stability and its interaction with PD-1 by inhibiting serine/threonine phosphorylation of PD-L1 by GSK3β, thereby preventing its degradation. This glycosylation does not affect PD-L1 acetylation or nuclear translocation. EGF-induced ubiquitination leads to increased mono- and polyubiquitination of glycosylated PD-L1. In addition, EGF stimulated upregulated PD-L1 phosphorylation, acetylation, but does not affect its SUMOylation in A431 cells. Palmitoylation of PD-L1 further enhances its stability by preventing ubiquitination and subsequent lysosomal degradation, highlighting the complex post-translational regulation of PD-L1 that balances its degradation and stability to control immune evasion mechanisms in cancer cells [13, 14, 73, 86, 117, 128].

**Fig. 4:**
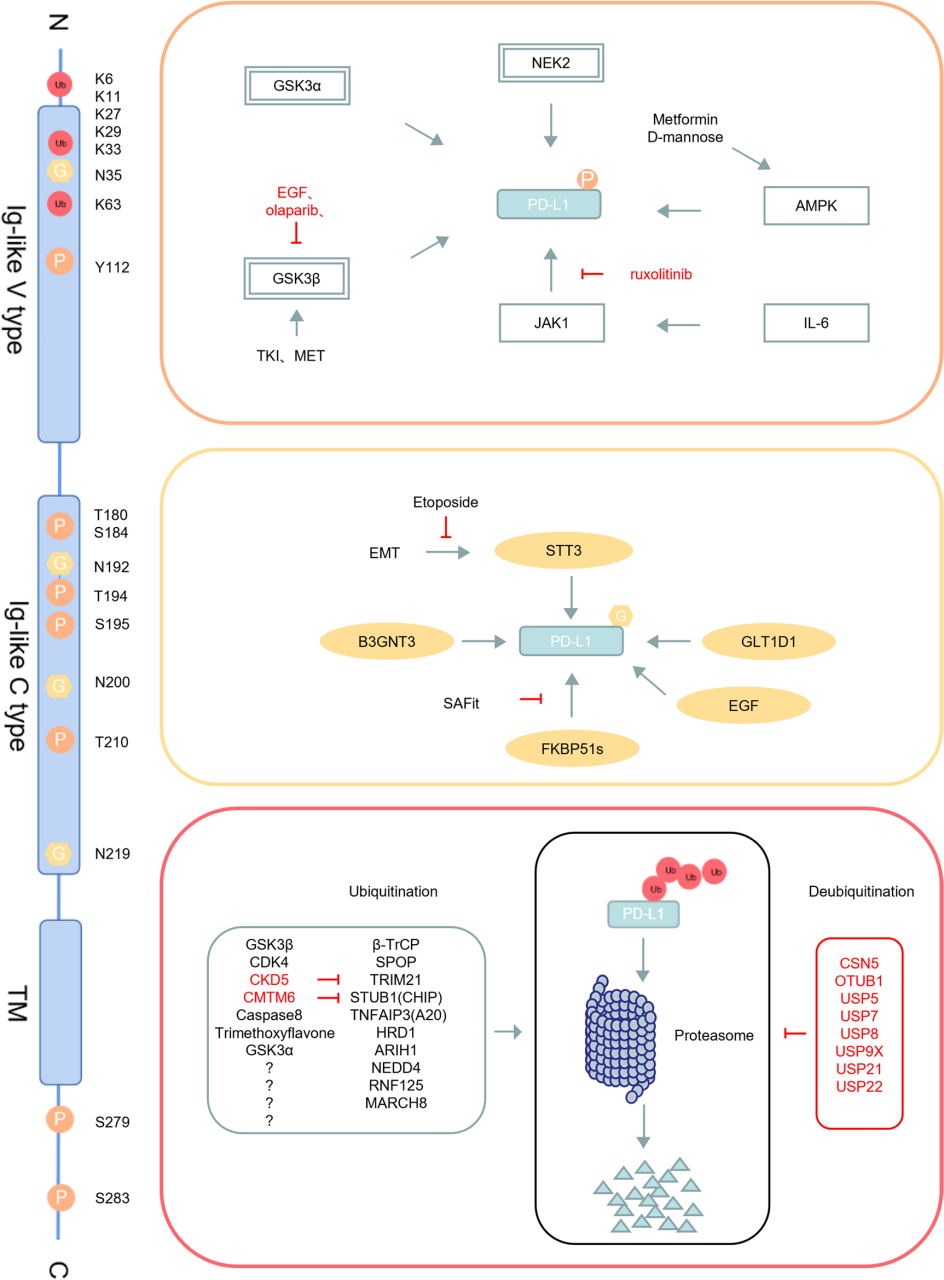
Structure and PTMs of PD-L1 (phosphorylation, glycosylation and ubiquitination): Presented here is a schematic diagram that illustrates the functional domains of PD-L1 as well as its diverse PTMs. PD-L1 is composed of two extracellular immunoglobulin-like domains, a transmembrane (TM) region, and an intracellular domain (ICD), and is regulated by multiple PTMs, including N-glycosylation (G, yellow hexagons), phosphorylation (P, orange circles), and ubiquitination (Ub, red circles). The corresponding modified amino acid residues, upstream regulators, and the outcomes of each post-translational modification are indicated in the figure. PD-L1 can be phosphorylated by GSK3α, GSK3β, NEK2, AMPK, and JAK1. STT3, B3GNT3, GLT1D1, EGF and FKBP51s can promote the glycosylation of PD-L1. PD-L1 can be polyubiquitinated by E3 ubiquitin ligases β-TRCP, SPOP, TRIM21, STUB1, TNFAIP3, HRD1, ARIH1, NEDD4, RNF125, and MARCH8. PD-L1 can be deubiquitinated by deubiquitinating enzymes CSN5, OTUB1, USP5, USP7, USP8, USP9X, USP21, and USP22. GSK3β, CDK4, Caspase8, Trimethoxyflavone, and GSK3α, as the corresponding upstream pathways, can promote the ubiquitination of PD-L1 mediated by the corresponding E3 ubiquitin ligases, while CDK5 and CMTM 6 respectively inhibit the ubiquitination of PD-L1 mediated by TRIM21 and STUB1. *GSK3* Glycogen synthase kinase 3, *STAT3* Signal Transducer and Activator of Transcription 3, *JAK1* Janus kinase 1, *AMPK* AMP-activated protein kinase, *IL-6* Interleukin-6, *B3GNT3* β-1,3-N-acetylglucosaminyltransferase 3, *EMT* Epithelial—Mesenchymal Transition, *β-TRCP* β-transducin repeat-containing protein, *SPOP* Speckle-type POZ protein, *TRIM21* Tripartite Motif Containing 21, *STUB1* STIP1 homology and U-Box containing protein 1, *TNFAIP3* Tumor necrosis factor-α-induced protein 3, *HRD1* HMG-CoA reductase degradation 1, *ARIH1* Ariadne RBR E3 ubiquitin protein ligase 1, *NEDD4* Neural precursor cell expressed, developmentally down-regulated 4, *RNF125* Ring Finger Protein 125, *MARCH8* Membrane-Associated Ring Finger (C3HC4) 8, *CSN5* COP9 signalosome subunit 5, *OTUB1* Ovarian tumor domain-containing ubiquitin aldehyde-binding protein 1, *USP* Ubiquitin Specific Protease, *CDK* Cyclin Dependent Kinase, *Caspase* Cysteine-aspartic acid proteas

Phosphorylation also plays a pivotal role in regulating PD-L1 stability. IL-6-activated JAK1 phosphorylates PD-L1 at Y112, which recruits the ER-associated N-glycosyltransferase STT3A, catalyzing PD-L1 glycosylation and preserving its stability [82]. In the absence of glycosylation, PD-L1 is phosphorylated at the T180 and S184 sites, leading to β-TrCP-mediated ubiquitination and degradation [141]. What’s more, AMPK phosphorylates PD-L1 at S195,which triggers abnormal glycosylation, resulting in PD-L1's degradation and increasing the anti-tumor activity of CTL [78]. Furthermore, kinases such as NEK2 and GSK3β have opposing effects on PD-L1 stability. NEK2 phosphorylates PD-L1 at T194 and T210, preventing its degradation mediated by ubiquitination modification, thereby upregulating PD-L1 expression [80]. The interplay between these kinases and their competing roles in regulating PD-L1 underscores the complexity of its PTMs, necessitating further research to fully understand how these processes are coordinated and whether additional kinases may also contribute to PD-L1's regulation (Fig. 4).

#### PD-L2

PD-L2, a crucial member of the B7 family, plays an important role in immune evasion by contributing to T cell dysfunction and helping cancer cells escape immune surveillance. As a ligand for PD-1, the biological significance of PD-L2 and the relationship between PD-L2 and its target molecules remain unclear, unlike PD-L1, which has been extensively studied and widely used, but PD-L2 has been shown to have overlapping functions with PD-L1 [55]. Glycosylation at specific sites, such as N157, N163, and N189, significantly enhances PD-L2's stability by preventing ubiquitin-mediated degradation [142]. Inhibition of N-linked glycosylation in colorectal cancer cells has been shown to markedly reduce PD-L2 protein levels, emphasizing the importance of glycosylation in maintaining PD-L2's stability [143]. Additionally, glycosylation prevents ubiquitin-dependent lysosomal degradation, thereby facilitating PD-L2's binding to PD-1, which promotes immune evasion. Recent studies have shown that EGF/STAT3 signaling drives PD-L2 glycosylation through the upregulation of the N-glycosyltransferase FUT8, a crucial enzyme in this process. This glycosylation mechanism is essential for enhancing tumor immune suppression and influencing the response to anti-EGFR therapies [144]. Inhibiting STAT3 with Stattic, a specific inhibitor, reduces PD-L2 glycosylation, promotes its degradation, and decreases its surface expression, leading to a lower affinity for PD-1 and the reactivation of T cell-mediated immune responses. Moreover, glycosylation at specific sites such as N64, located in the C-D loop region of PD-L2, has been shown to enhance the dynamic properties of this region, thereby affecting PD-1/PD-L2 dissociation. Inhibition of glycosylation at the N64 site increases PD-L2 binding affinity for PD-1, indicating potential therapeutic implications for cancer immunotherapy [145]. Although glycosylation sites at N10 and N43 have also been identified, their functional significance remains unclear [146].

Overall, these findings underscore the importance of glycosylation in regulating PD-L2's stability and function, offering new avenues for therapeutic interventions aimed at modulating PD-L2's interactions with PD-1 to improve cancer immunotherapy outcomes.

### CD47

**Fig. 5:**
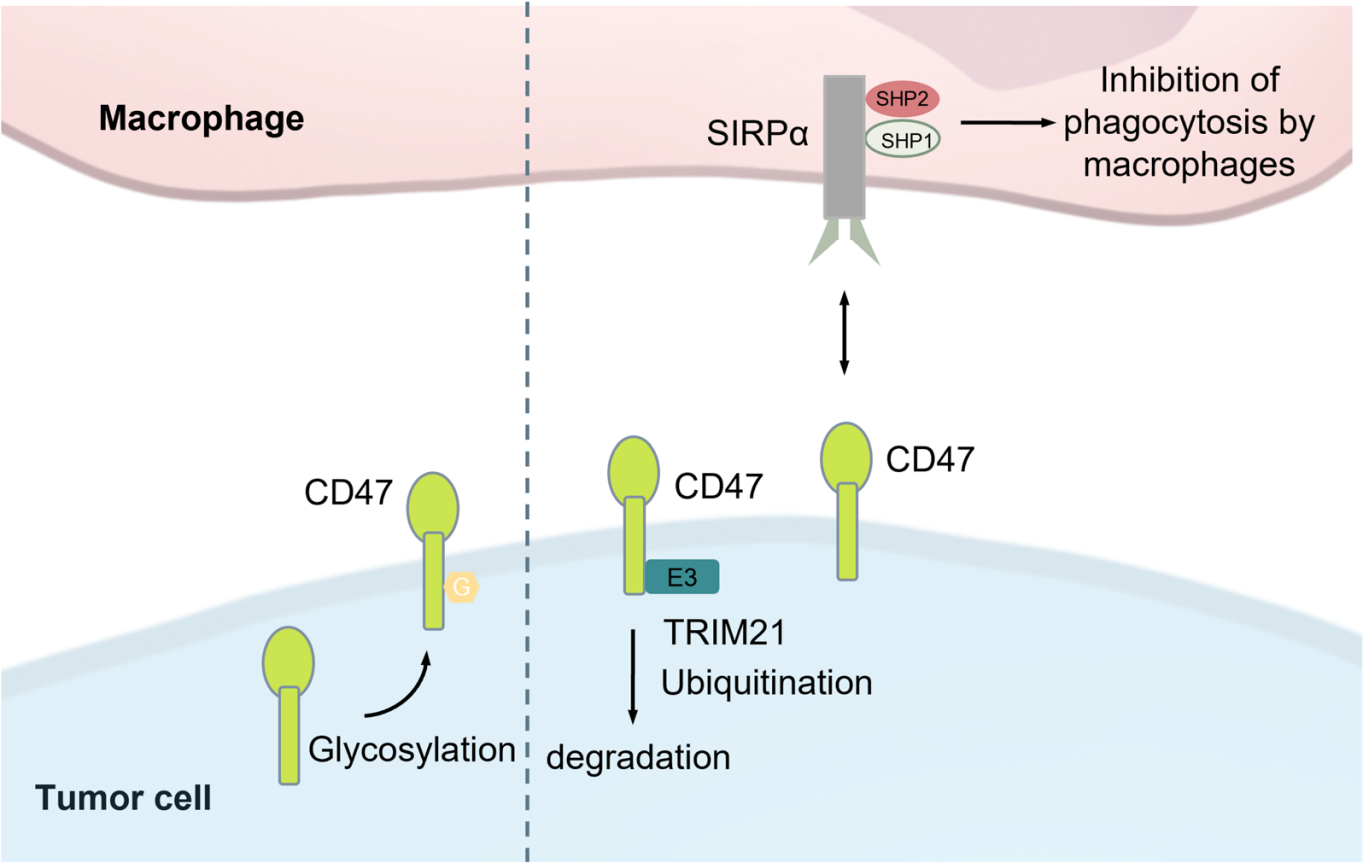
PTMs of CD47: When CD47 on the surface of tumor cells interacts with SIRPα on macrophages, it causes the intracellular portion of SIRPα to be phosphorylated. Then, SHP-1 and SHP2 are activated, which subsequently inhibits myosin IIA and prevents the phagocytosis of macrophages. There are six potential N-glycosylation sites on CD47, but whether glycosylation affects the binding of CD47 to its ligands, as well as the ligand-binding characteristics of CD47 and subsequent signal transduction, remains highly uncertain. CD47 can be ubiquitinated by the E3 TRIM21 ligase and then transported to the proteasome for degradation, thereby blocking the CD47/SIRPα signaling pathway and enhancing anti-tumor immunity. *SIRPα* Signal Regulatory Protein Alpha, *CD47* Cluster of Differentiation 47, *SHP1* Src homology region 2 (SH-2) domain-containing phosphatase 1, *SHP2* Src homology region 2 (SH-2) domain-containing phosphatase 2, *TRIM21* tripartite -motif protein 21

CD47, also known as Integrin-associated protein (IAP), is the first identified phagocytosis checkpoint and is structurally characterized as a transmembrane glycoprotein with glycosylation [147, 148]. The primary ligand for CD47 is SIRPα (also known as BIT, SHPS-1, and CD172a), which, upon binding to CD47, recruits and activates intracellular SHP1 and SHP2 proteins. This interaction initiates a series of downstream cascades that lead to the dephosphorylation of Myosin IIA, inhibit cytoskeletal rearrangement, and ultimately emit a "Don't eat me" signal, thereby preventing macrophage-mediated phagocytosis and facilitating immune evasion [149–151]. In addition to SIRPα, other ligands such as thrombospondin-1 (TSP-1), integrin α2β1, and αvβ3 have also been identified as CD47 binding partners [152–157]. Through interactions with these ligands, CD47 regulates various cellular functions by activating downstream phosphorylation signaling pathways (Fig. 5). However, further studies are needed to explore these pathways and their potential as targets for antitumor therapy. Although CD47 has been established as a key regulator of intracellular phosphorylation signaling [158–160], the specific mechanisms by which CD47 itself may undergo phosphorylation modifications remain to be elucidated.

Six potential N-glycosylation sites have been identified on CD47 [157]; however, it remains uncertain whether glycosylation affects CD47's ligand-binding properties and its subsequent signaling pathways. A study by Ranganath et al. (2006) demonstrated that glycosylation is essential for anchoring CD47 to the cell membrane in a yeast model [161], implying that glycosylation might influence CD47's ligand-binding capacity. Contrarily, Winston and Shyamsundar et al. reported that although both CD47 and SIRPα undergo post-translational glycosylation, glycosylation is not required for their interaction [162, 163]. Further supporting this, recombinant monomeric CD47 and SIRPα expressed in E. coli were shown to disrupt the CD47-SIRPα interaction in vitro, despite lacking glycosylation, indicating that glycosylation is not necessary for this interaction [164]. Although the precise role of CD47 glycosylation remains unclear, different glycosylation patterns of CD47 in malignant versus normal cells have been observed. This differential glycosylation has led to the development of a bispecific antibody, TJC4, which selectively binds to tumor cells without cross-reacting with CD47 on erythrocytes. This suggests that targeting glycosylation modifications may provide a promising strategy for the development of CD47-related biotherapeutic drugs [165–167].

Current research on CD47 ubiquitination is limited, though some studies suggest a potential role for ubiquitin-associated protein PLIC-1 in interacting with CD47, possibly contributing to the integration of adhesion and signaling components involved in cell migration [168, 169]. Another recent study [89] demonstrated that in the post-translational regulation of CD47, ubiquitination mediated by the E3 ligases TRIM21, facilitates CD47 degradation. Additionally, bioinformatics analysis using the UbiBrowser platform (http://ubibrowser.ncpsb.org/) identified five E3 ligases predicted to target CD47 with moderate confidence. These findings suggest the potential for developing small molecule inhibitors based on PROTAC (proteolysis-targeting chimera) technology. Zhang et al. engineered checkpoint nano-proteolysis targeting chimeras (nano-PROTACs) that induce targeted degradation of SHP2 via the UPS. This strategy blocks checkpoint signaling pathways, including CD47/SIRPα and PD-1/PD-L1, thereby activating cancer photoimmunotherapy and reinvigorating antitumor macrophages and T cells [170].

### CTLA-4

**Fig. 6:**
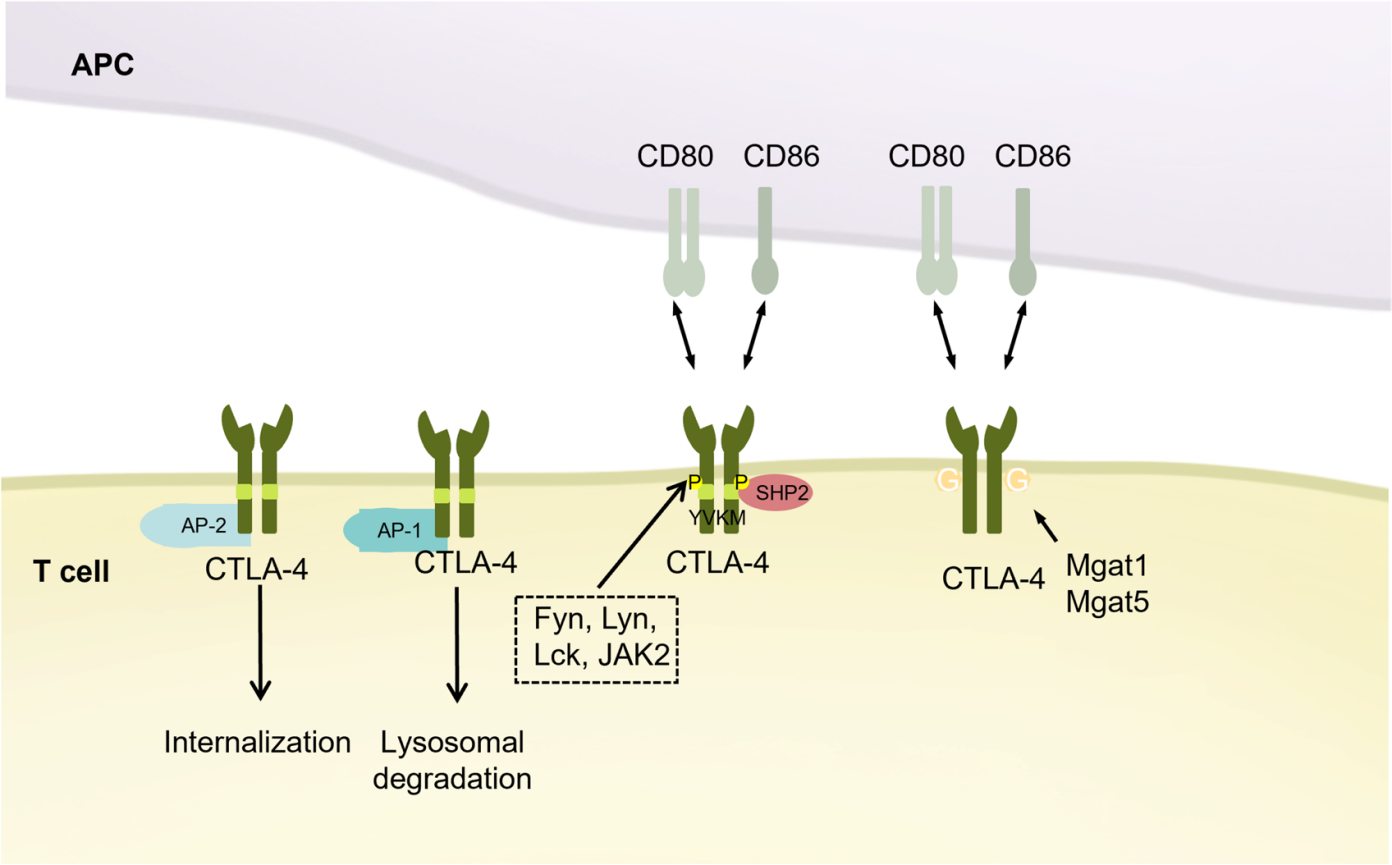
PTMs of CTLA-4: CTLA-4 binding to CD80/86 affects T cell function. Phosphorylation of CTLA-4 YVKM motifs recruits SHP2. The clathrin adaptor complex AP-1 binds to CTLA4, contributing its entry into the lysosome for degradation. In addition, CTLA-4 binds to the AP-2, and consequently undergoes AP-2-mediated internalization. Fyn, Lyn, Lck, JAK2 can promote CTLA4 phosphorylation. Mgat1 and Mgat5 promotes the surface retention of CTLA-4 through the regulation of glycosylation. *CTLA-4* Cytotoxic T-lymphocyte–associated antigen 4, *SHP2* Src homology region 2 (SH-2) domain-containing phosphatase 2, *CD80* Cluster of Differentiation 80, *CD86* Cluster of Differentiation 86, *Fyn* FYN Proto-Oncogene, *Lyn* LYN Proto-Oncogene, *Lck* LCK Proto-Oncogene, *JAK2* Janus Kinase 2, *Mgat1* Alpha-1,3-Mannosyl-Glycoprotein 2-Beta-N-Acetylglucosaminyltransferase, *Mgat5* Alpha-Mannoside Beta-1,6-N-Acetylglucosaminyltransferase V

Cytotoxic T lymphocyte-associated protein 4 (CTLA-4/CD152), a type of transmembrane protein that generates T cell inhibitory signals by binding to ligands [171]. It is predominantly expressed by T cells, particularly regulatory T cells (Tregs). CTLA-4 shares significant homology with the T cell co-stimulatory molecule CD28 [172], and both CTLA-4 and CD28 interact with CD80 (B7-1) and CD86 (B7-2, B70) on antigen-presenting cells (APCs) to initiate co-stimulatory or co-inhibitory signaling pathways (Fig. 6) [173, 174]. Although CTLA-4 is primarily localized intracellularly, it translocated to the cell surface upon T cell activation, where it exerts its inhibitory function [175]. Notably, even at low surface expression levels, CTLA-4 can still mediate inhibitory signaling, leading to the hypothesis that intracellular CTLA-4 also plays a crucial role in T cell inactivation [176].

Phosphorylation plays a crucial role in determining the localization and signaling of CTLA-4. Tyrosine residues Y165, Y182, Y201, and Y218 have been identified as phosphorylation sites on CTLA-4. The YVKM motif (Y165) was the first confirmed site, and early studies revealed that the phosphorylation status of this motif influences the trafficking, internalization, and function of CTLA-4 [177, 178]. In the absence of Y165 phosphorylation, CTLA-4 binds to the clathrin adaptor complex AP-2, leading to internalization mediated by AP-2 [178, 179]. Conversely, phosphorylation of Y165 stabilizes CTLA-4 at the cell surface, facilitating CD80/86 binding and initiating inhibitory signaling [180]. More recent studies have confirmed that the YVKM motif is critical for regulating CTLA-4 internalization [181].

However, Nakaseko et al. demonstrated that the inhibition of T-cell activation by CTLA-4 was independent of the YVKM motif and that its inhibitory function did not require tyrosine phosphorylation. This suggests that CTLA-4 operates on two distinct regulatory levels: a phosphotyrosine-dependent mechanism for surface retention and a phosphotyrosine-independent interaction with signaling molecules [176, 182]. As research has progressed, this contradiction persisted, with Schneider et al. reporting that the presence of the YVKM motif helps block TCRζ signaling, or combined TCRζ/CD28 signaling. Schneider's study attributed the discrepancy to differences in antibody presentation—soluble versus immobilized—indicating that soluble antibodies are more dependent on the YVKM motif than immobilized ones [183, 184]. Additionally, Schneider et al. identified another clathrin adaptor complex, AP-1, which also binds to the GVYVKM motif but shuttles CTLA-4 from the trans-Golgi network (TGN) to the lysosome for degradation [185].

In characterizing the kinases responsible for CTLA-4 tyrosine phosphorylation, researchers have identified members of the Src family—Fyn, Lyn, and Lck—as key players that bind to CTLA-4 and phosphorylation at the Y165 and Y182 sites. This phosphorylation induces CTLA-4 to recruit the tyrosine phosphatase SHP2 in a Fyn-dependent manner [186]. Conversely, Chuang et al. demonstrated that Fyn and Lck also phosphorylate the Y201 and Y218 sites of CTLA-4, and they found that phosphorylation at Y201 facilitates CTLA-4 recruitment of SHP2. Their findings also indicated that Y201 phosphorylation is correlated with CTLA-4 accumulation on the cell surface [187]. In contrast to Y201, Baroja et al. showed that ZAP-70 promotes the cell surface retention of CTLA-4 by inducing phosphorylation at the Y165 and Y182 sites [176]. Further investigations by Hu et al. found that the transfection of Fyn or Lck can enhance the phosphorylation of intracellular of CTLA-4 and facilitate the recruitment of PI3K [188]. In addition to Fyn, Lyn, and Lck, studies discovered that the tyrosine kinase JAK2 phosphorylates Y165 in HUT78 T cell lines [189]. It was also observed that depletion of PAG enhances Src kinase activity and proximal T cell receptor signaling, causing T-cell unresponsiveness; this is achieved by Fyn-dependent hyperphosphorylation of CTLA-4, which in turn leads to the recruitment of SHP-1 into lipid rafts [190]. Despite the extensive literature on CTLA-4 phosphorylation, the conclusions remain inconsistent and sometimes contradictory. Thus, further precise studies are necessary to clarify the role and implications of these phosphorylation modifications.

The internalization and surface retention of CTLA-4 are regulated not only by tyrosine phosphorylation but also by N-glycosylation [191], with N-acetylaminoglucosyltransferase I (Mgat1) playing a critical role in this process. Treatment with Vitamin D3 has been shown to enhance Mgat1 expression and increase N-glycan chain branching, which results in reduced internalization and elevated surface levels of CTLA-4 in T cells [192]. Furthermore, TCR signaling promotes the N-glycosylation of CTLA-4 by enhancing glucose uptake and upregulating Mgat5, thereby maintaining CTLA-4 on the cell surface [193]. Additionally, the common CTLA-4 polymorphism T17A is correlated with changes in CTLA-4 glycosylation levels. Aberrant glycosylation of the CTLA-4Ala^17^ variant has been linked to inhibited CTLA-4 surface expression [194].

N-glycosylation is also essential for CTLA-4 signaling. Structural analyses have suggested that N-glycosylation may indirectly mediate the interaction between CTLA-4 and its ligands, CD80/CD86 [195], by maintaining the proper orientation or spatial organization of CTLA-4 [196, 197]. Two N-linked glycosylation sites, N78 and N110, have been identified as critical for CTLA-4 to form dimers [198]. Metzler et al. demonstrated that the N78 glycosylation site is crucial for ligand binding, as the N78D mutation led to significant aggregation and loss of CD80/CD86 binding, whereas the mutation of N110 had no such effect [196]. Moreover, Dong et al. (2020) used a humanized CTLA-4 antibody, mAb146, to show that the conserved N110 glycosylation site plays a role in cross-species binding and the functional of the antibody [199]. In addition to N-glycosylation, advancements in high-resolution mass spectrometry have revealed unexpected O-linked glycosylation in CTLA-4 Fc-fusion proteins. This O-linked glycosylation deficiency likely reduces protein aggregation, possibly by limiting the presence of junctional O-glycans that impede interchain disulfide bond reformation [200].

The ubiquitination of CTLA-4 remains largely unexplored, with both the specific E3 ligase responsible for CTLA-4 ubiquitination and the ubiquitination site on CTLA-4 yet to be identified. Although direct ubiquitination modifications of CTLA-4 have not been fully characterized, studies on CTLA-4-deficient T cells have shown a significant reduction in overall ubiquitination levels, suggesting the crucial role of ubiquitination in the CTLA-4 pathway [201].

### B7-H3

B7-H3 (CD276), a member of the B7 immunoglobulin superfamily, is a type I transmembrane protein that is notably overexpressed in various solid tumors, including bladder cancer, prostate cancer, and melanoma, while exhibiting limited expression in normal tissues. B7-H3 primarily functions as an immunoinhibitory molecule, facilitating immune evasion by tumor cells. Beyond its immune checkpoint role, B7-H3 is implicated in promoting tumor cell migration, proliferation, invasion, angiogenesis, and drug resistance [202].

B7-H3 is a highly glycosylated protein, with current studies identifying eight glycosylation sites: N91, N104, N189, N215, N309, N322, N407, and N433 [203]. In triple-negative breast cancer (TNBC), where PD-1/PD-L1 immunotherapy often proves ineffective, recent research has uncovered a novel mechanism by which core fucosylation of B7-H3 at its N-glycans facilitates immune escape. Targeted intervention aimed at inhibiting B7-H3 core fucosylation enhances TNBC cell sensitivity to PD-L1 mAb therapy. Mechanistic studies reveal that this core fucosylation is mediated by FUT8, which stabilizes B7-H3 and maintains its presence on the cell membrane. Knockdown of FUT8, which inhibits glycosylation, reduces B7-H3-mediated immunosuppressive functions [203]. Additionally, research on Ca9-22 oral cancer cells demonstrated that B7-H3 glycan contains terminal α-galactose and exhibits higher fucosylation and enhanced interactions with immune cells compared to B7-H3 from normal SG cells. This abnormal glycosylation profile of B7-H3 presents potential diagnostic and therapeutic opportunities for oral cancer [204]. Furthermore, in esophageal squamous cell carcinoma (ESCC), investigators found that B7-H3 glycosylation, particularly fucosylation, is significantly upregulated, promoting the development and progression of ESCC and suggesting that B7-H3 fucosylation may serve as a potential biomarker for this cancer [205].

In addition to glycosylation, B7-H3 has been identified to undergo phosphorylation and ubiquitination modifications at specific sites—S513 and T551 for phosphorylation, and K521 and K526 for ubiquitination. However, the functional roles of these PTMs in the regulation of B7-H3 have not yet been thoroughly investigated.

### TIM-3

**Fig. 7:**
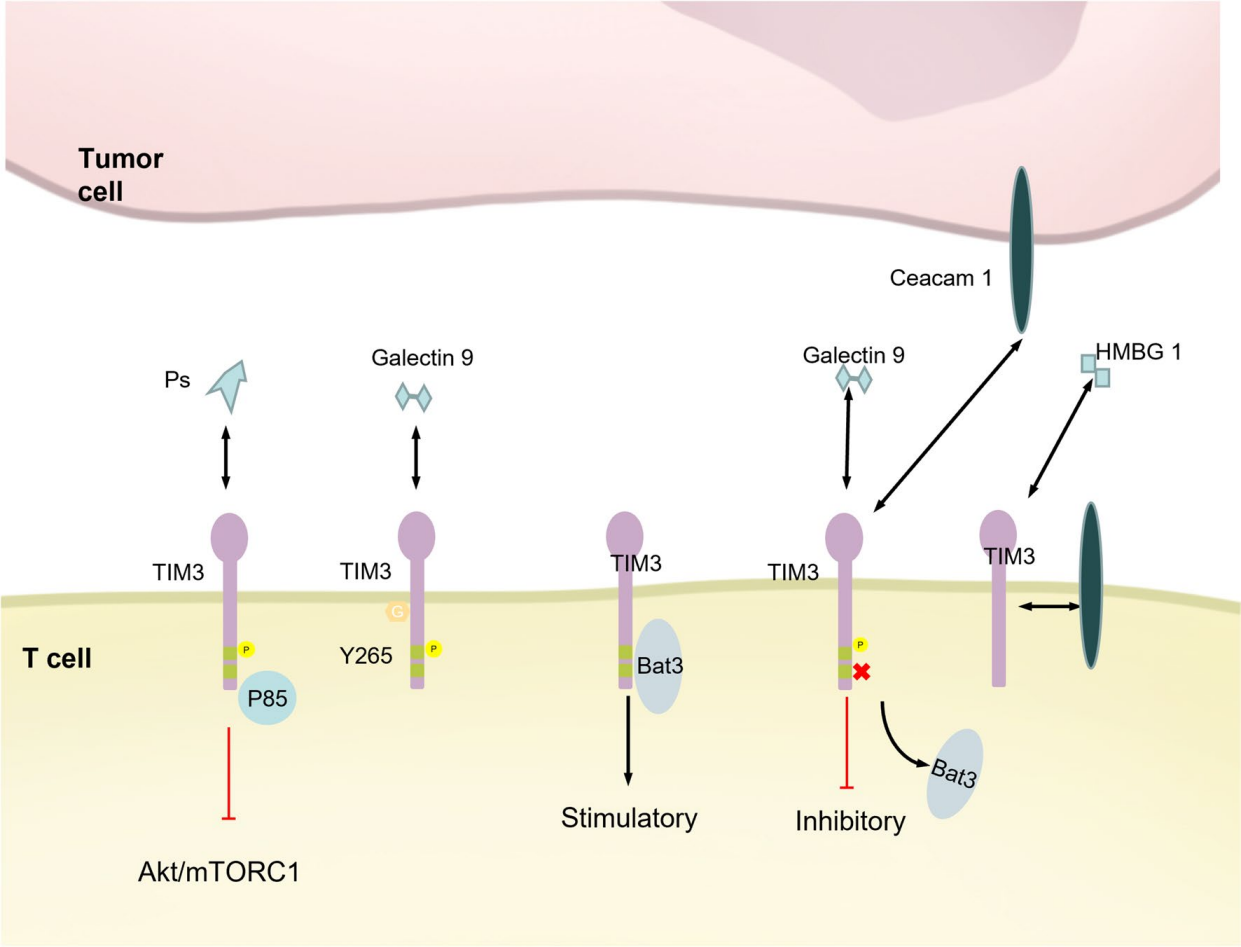
PTMs of TIM3: TIM3 has four known ligands: HMBG1, Ceacam1, PS, and Galectin9. Bat3 binds to the unphosphorylated Y256/263 sites of TIM3's cytoplasmic domain to send stimulatory signals in T cells. Interaction with Galectin9/Ceacam1 phosphorylates TIM3's Y265 (Y256 in mouse) and Y272 (Y263 in mouse) sites, preventing Bat3 binding and turning TIM3 from stimulatory to inhibitory. PS binds to TIM3 and promotes its phosphorylation, thereby inhibiting AKT/mTOR signaling. TIM3 can also be glycosylated, and its mechanism and function are unknown. *mTORC* Mammalian target of rapamycin complex, *Bat3* HLA-B Associated Transcript 3, *TIM-3 (HAVCR2)* T cell immunoglobulin and mucin domain-containing molecule 3, *Ps* Phosphatidylserine, *HMGB1* High mobility group box 1 protein, *Ceacam1* CEA Cell Adhesion Molecule 1

TIM-3 (HAVCR2) is a one of the key immune checkpoints, initially identified on the surface of Th1 cells, where it functions as an activation-induced suppressor molecule. It plays a key role in mediating immune tolerance, particularly in the context of chronic viral infections and cancer [206]. TIM-3 has four known ligands: the galectin-9, carcinoembryonic antigen-related cell adhesion molecule 1 (Ceacam1), high mobility group box-1 protein (HMGB1), and the non-protein ligand PS [207]. Studies indicate that TIM-3 is a marker of T cell exhaustion, and therapeutic targeting of TIM-3 has shown promise in rejuvenating T cells, enabling them to persist in combating pathogens or tumor cells **(**Fig. 7**)**.

TIM-3 contains five conserved tyrosine residues in its cytoplasmic region, among which Y265 and Y272 play an extremely crucial role in regulating T-cell signaling and can be phosphorylated by kinases such as ITK or Src family kinase [208–210]. In the absence of phosphorylation, the protein BAT3 binds to TIM-3, inhibiting TIM-3-induced T cell exhaustion and death. Phosphorylation of Y265 and Y272, however, causes BAT3 dissociation, reversing its inhibitory effects [211]. Galectin-9 has been reported to promote phosphorylation of the Y265 site [209, 212], indicated that the tyrosine residue is important for regulating T-cell fate. Additionally, Leishmania donovani recruits and activates the non-receptor tyrosine kinase Btk by promoting TIM-3 phosphorylation in dendritic cells (DCs). This activation of Btk, in turn, inhibits DC activation/maturation by suppressing the NF-κB pathway in a manner that depends on IL-10 [213]. Earlier structural studies revealed that phosphatidylserine binds to TIM-3 to promote salt bridge formation and the release of tyrosine-containing chain [214]. More recent research has shown that PS can also promote TIM-3 phosphorylation, which competes with PI3K p110 for binding to P85, resulting in the inhibition of downstream Akt/mTORC1 signaling and dysfunction in two natural killer (NK) cell subpopulations [215]. Building on this, researchers developed a phosphatidylserine photoswitch element (phoPS) that modulates TIM-3 phosphorylation and NK cell function in a light-dependent manner [216].

Glycosylation may be crucial for TIM-3, as the binding activity of galectin-9 to TIM-3-Ig fusion protein is influenced by N-glycosylation [217]. Additionally, iso- and heterotypic interactions between TIM-3 and other TIM family proteins are also dependent on glycosylation modifications [218]. Computational modeling has suggested that glycosylation at the N78 site may affect the binding of TIM-3 to small molecule ligands, providing a potential avenue for designing small molecule drugs targeting TIM-3 [219]. The NetOGlyc 4.0 algorithm predicts that TIM-3 has eight O-glycosylation sites; however, the functions of these glycosylation sites remain unexplored [220]. Moreover, studies have indicated that N-glycosylation site mutants, such as N53Q and N100Q, do not impair TIM-3 binding to ligands on CD4( +) CD25( +) T cells [217].

### TIGIT

**Fig. 8:**
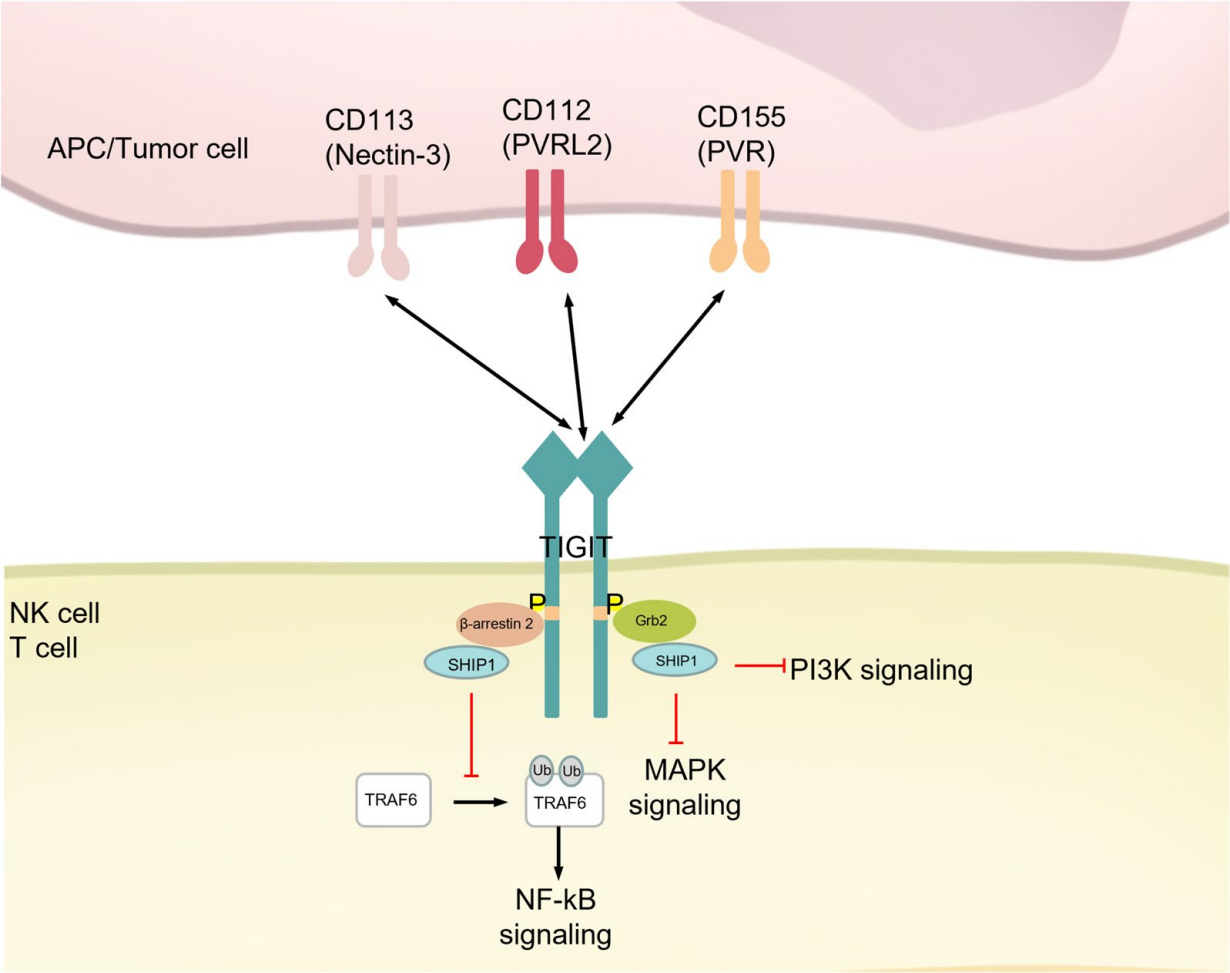
PTMs of TIGIT: TIGIT binds to CD112, CD113, and CD155. TIGIT inhibits the PI3K, MAPK, and NF—κB signaling pathways by recruiting SHIP1. *TIGIT* T cell immunoreceptor with Ig and ITIM domains, *Grb2* Growth factor receptor-bound protein 2, *β-arrestin 2* Beta-arrestin 2, *SHIP* Src homology 2 domain-containing inositol 5-phosphatase, *TRAF6* Tumor necrosis factor receptor-associated factor 6, *NF-kB* Nuclear factor kappa-light-chain-enhancer of activated B cells, *MAPK* Mitogen-activated protein kinase, *PI3K* Phosphoinositide 3-kinase

The T cell immunoreceptor with Ig and ITIM domains (TIGIT), also known as VSIG9, VSTM3, and WUCAM, is a member of the CD28 family that consists of an extracellular immunoglobulin variable domain, a type I transmembrane domain, and an intracellular domain [221]. It is expressed on T cells and NK cells, with its expression being upregulated upon cell activation. Functioning as a co-inhibitory receptor, TIGIT binds to several ligands including CD155 (poliovirus receptor, PVR), CD112 (PVRL2, nectin-2), and CD113 (nectin-3). This binding inhibits the cytotoxic activities of T cells and NK cells, positioning TIGIT as a novel immune checkpoint following the PD-1/PD-L1 pathway (Fig. 8) [222, 223].

Current research on TIGIT signaling has primarily focused on NK cells, highlighting the significance of phosphorylation modifications in the ITIM and the ITT-like motif within the cytoplasmic domain of TIGIT. In murine models, phosphorylation of ITIM at Y233 and the ITT-like motif at Y227 has been shown to trigger inhibitory signaling [224]. In human NK cells, binding of TIGIT to its ligand, poliovirus receptor (PVR), induces phosphorylation at the Y225 site within the ITT-like motif. This phosphorylation event facilitates the recruitment of GRB2 and β-arrestin 2, which, through the involvement of SHIP-1, inhibit PI3K and MAPK signaling pathways as well as TRAF6-mediated NF-κB activation, ultimately leading to functional inhibition of NK cells [225, 226]. The Y231 site within the ITIM of human TIGIT also undergoes phosphorylation, although its role remains contentious. Noa et al. suggest that phosphorylation at Y231 mediates inhibitory signaling, while Liu et al. report that mutations in Y231 result in only minor effects on NK cell function [223, 226]. In addition to phosphorylation, recent studies have drawn attention to the glycosylation of TIGIT. Specifically, N-glycosylation at the N32 and N101 sites has been identified as crucial for modulating TIGIT's binding affinity to PVR, making these sites potential targets for future immunotherapeutic strategies [227].

### LAG-3

**Fig. 9:**
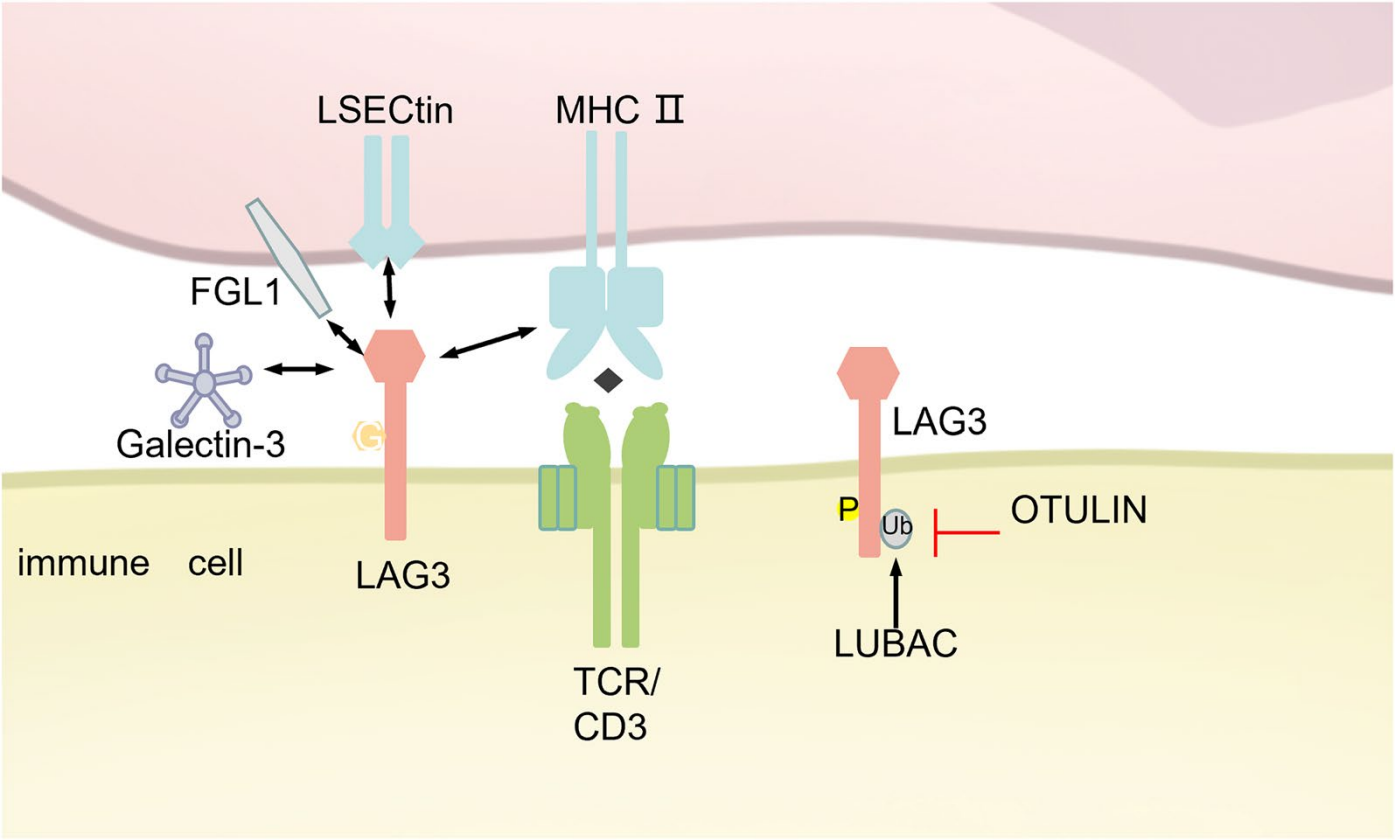
PTMs of LAG3: LAGs binds to LSECtin, FGL1, Galectin-3, MHC-II. LAG3 was found to have ubiquitination, phosphorylation, and glycosylation modification sites. However, only a few ubiquitination-related mechanisms have been studied. Currently, LUBAC is found to promote LAG3 ubiquitination, while OTULIN inhibits LAG3 ubiquitination. *LAG3* Lymphocyte-activation gene 3, *LCK* Lymphocyte-specific protein tyrosine kinase, *LSECtin* Liver sinusoidal endothelial cell lectin, *MHC* Major histocompatibility complex, *LUBAC* linear ubiquitin chain assembly complex, *FGL1* fibrinogen-like protein 1, *OTULIN* OTU deubiquitinase with linear linkage specificity

Lymphocyte activation gene-3 (LAG-3, CD223) is an activation-induced cell surface molecule that belongs to the immunoglobulin superfamily (IgSF) [228]. LAG-3 has been found to be expressed on a variety of immune cells, including T cells, NK cells, plasmacytoid dendritic cells (pDCs), and B cells [229, 230]. Structurally, LAG-3 shares similarities with CD4, as both proteins contain four conserved extracellular immunoglobulin-like domains. Notably, LAG-3 binds to MHC class II molecules with higher affinity than CD4 [231]. Beyond MHC-II, LAG-3 also interacts with other ligands such as galectin-3, LSECtin, and fibrinogen-like protein 1 (FGL1) (Fig. 9) [232–234]. Although LAG-3 has been shown to negatively regulate T cell activation and proliferation, the precise signaling mechanisms involved remain unclear.

LAG-3 is a glycoprotein with four potential N-linked glycosylation sites within its extracellular domain. Studies showed that the binding of MHC class II and FGL1 to LAG-3 is dependent on glycosylation [233–235]. However, beyond this, the impact of glycosylation on LAG-3 function remains largely unexplored. Regarding phosphorylation, human LAG-3 possesses only two serine residues, S484 and S497, and lacks threonine and tyrosine residues. Studies indicate that single or double mutations inactivating these serine phosphorylation sites do not affect the protein kinase C (PKC) signaling-mediated translocation of LAG-3 to the cell membrane [236]. In the context of ubiquitination, LAG-3 has been identified as a substrate for the linear ubiquitin chain assembly complex (LUBAC) and the DUB OTULIN. Exogenous ubiquitination assays have demonstrated that the HOIP component, along with HOIL-1L, mediates the ubiquitination of LAG-3, whereas OTULIN facilitates its deubiquitination. Further analysis revealed that mutations such as K498R, 3KR (K356R/K366R/K498R), and LAG-3-K0 have minimal impact on LAG-3’s overall ubiquitination levels. However, when combined with serine mutations (LAG-3-K0-S484A or LAG-3-K0-S497A), a significant reduction in LAG-3 ubiquitination was observed [237].

## Clinical applications of combined ICI therapies with PTMs targeted therapy

### The current status and dilemmas of immune checkpoint therapy

ICB therapy has emerged as a more effective and durable treatment option for patients with advanced cancers compared to conventional therapies, resulting in favorable therapeutic outcomes. Since the FDA approval of the CTLA-4 antibody ipilimumab in 2011 for the treatment of advanced melanoma, several antibodies targeting CTLA-4, PD-1, and PD-L1 have been approved for various cancers. These include the CTLA-4 inhibitor ipilimumab, and PD-1/PD-L1 inhibitors such as pembrolizumab, nivolumab, atezolizumab, durvalumab, and avelumab. The indications for these therapies have expanded from melanoma and NSCLC to gastric cancer, hepatocellular carcinoma, head and neck cancer, renal cell carcinoma (RCC), bladder cancer, anal cancer, colorectal cancer, TNBC, and Hodgkin's lymphoma, among others [238–245]. Recent advancements in cancer immunotherapy have ushered in the era of immune checkpoint blockade, with newer targets such as CD47, LAG-3, TIM-3, TIGIT, and B7-H3 being explored in preclinical and clinical trials as potential therapeutic agents (Table 1). A search of ClinicalTrials.gov and the NCI Drug Dictionary reveals numerous ICIs undergoing clinical evaluation, many of which have demonstrated promising outcomes, suggesting these inhibitors hold potential for refining existing antitumor therapies. However, despite these successes, limitations in the use of ICIs have emerged during clinical application. These challenges include a low response rate in certain tumor types [246, 247], the occurrence of immune-related adverse events (irAEs) [248–250], and the development of resistance in some patients, leading to poor prognosis [251–255]. Resistance mechanisms to ICIs are complex, dynamic, and interconnected, and are classified as either primary or acquired resistance. Understanding and overcoming these resistance pathways is critical for improving the efficacy of ICB therapy. Several studies have shown that compared with monotherapy, the combination therapy of TIM3 or LAG3 inhibitors with PD-1 blockade therapy can significantly enhance anti-tumor activity, and patients have good tolerance to the combination therapy [256–260]. It is worth noting that in 2022, the FDA approved the combination of Relatlimab, a monoclonal antibody targeting LAG3, with nivolumab (an anti-PD-1 inhibitor) for the treatment of metastatic melanoma [261]. This emphasizes the potential of combination therapies and novel immune checkpoint blockade in overcoming acquired drug resistance and improving treatment outcomes for various cancers.

**Table 1:** The more promising immune checkpoints

Immunization checkpoints		Expression position	Ligands	Inhibitor	Number of clinical trials
CTLA-4	CD152	Activated T-cells, etc	CD80 CD86 (B7-1 and B7-2)	Ipilimumab, Tremelimumab	695
PD-1	CD279	Activated T-cells, etc	PD-L1 PD-L2	Keytruda,	3846
				Nivolumab (Opdivo), Pembrolizumab, Cymplimab, Toripalimab	
PD-L1	CD274、B7H1	Tumor cells, etc	PD-1	Atezolizumab (Tecentrip),	3003
				Durvalumab (Imfinzi),	
				Avelumab (Bavencio),	
PD-L2	B7DC, CD273	Dendritic cells, etc	PD-1	–	261
LAG-3	CD223	Activated CD4( +) and CD8( +) T cell subsets, etc	MHC-II, FGL1, Galectin-3, LESCtin	Relatlimab	155
TIM-3	HAVCR2	Activated T cells, B cells, etc	Galectin-9, Ceacam-1,	Cobolimab	106
			HMGB1, PtdSer		
TIGIT	WUCAM	T cells, NK cells, etc	CD155, CD112, CD113	Tiragolumab	88
CD47	IAP	Almost all normal cell surfaces	SIRPα, platelet protein-1 (TSP-1), integrin α2β1, αvβ3	Magrolimab (fail)	95
B7H3	CD276	Immune cell surfaces such as dendritic cells, monocytes, B-cells, etc	–	MGC-018 (Vobramitamab duocarmazine), DS-7300 (Ifinatamab Deruxtecan)	97

Primary resistance to immunotherapy is defined as the lack of an objective response of the primary tumor to treatment, despite the absence of prior exposure to ICIs [262]. The primary mechanisms contributing to this resistance include the absence of antigens on the tumor surface, which prevents immune recognition, and defects in antigen-presenting mechanisms that impair the immune system's ability to detect and respond to cancer cells [263]. Additional factors include poor infiltration of tumor-infiltrating lymphocytes (TILs) [251], a reduction in the number of DCs that are critical for initiating immune responses [264], and the activation of myeloid-derived suppressor cells (MDSCs) and tumor-associated macrophages (TAMs) [265, 266], which can create an immunosuppressive TME. Understanding these mechanisms is essential for developing strategies to overcome primary resistance and enhance the efficacy of immunotherapy.

Acquired resistance refers to the phenomenon where tumors initially respond to ICIs but later achieve immune homeostasis and eventually escape immune surveillance during treatment [262, 267]. The mechanisms driving acquired resistance include the depletion of tumor antigens, which reduces the immune system's ability to recognize and target cancer cells [268]. Additionally, tumors can produce immunosuppressive molecule, such as adenosine, which impair lymphocyte function and contribute to immune escape [269–271]. Furthermore, the upregulation of alternative immune checkpoints, such as LAG-3, TIGIT, B and T lymphocyte attenuator (BTLA), and TIM-3, can further suppress the immune response and enable tumor progression despite ongoing ICI therapy [271–273]. Understanding these mechanisms is crucial for developing strategies to counteract acquired resistance and improve long-term outcomes in cancer immunotherapy.

Combination therapies that integrate ICBs with other antitumor agents hold promise for overcoming the limitations of monotherapy, with the potential to enhance response rates, prolong the duration of response, and even activate antitumor immune memory. Notably, such combination strategies can reduce adverse effects and improve drug safety through dose adjustments without compromising therapeutic efficacy. Common approaches include combining ICBs with chemotherapeutic agents [254, 274–276], anti-angiogenic agents [277], IDO inhibitors [278, 279], molecular antibodies like Checkmate, and cell therapies [267] (NCT02998528, NCT03778814, NCT02742727).

### The advantages of targeting PTMs in combination with immune checkpoints

However, current clinical evidence indicates that the outcomes of these combination strategies often do not meet expectations, underscoring the need for a deeper understanding of ICI resistance mechanisms and the development of new drugs to enhance ICI efficacy. Increasingly, research is highlighting the pivotal role of PTMs in regulating immune checkpoints during tumor therapy. Various PTMs, including phosphorylation, glycosylation, ubiquitination are closely linked to immune cell activation, signal regulation, immune response, and tumor metabolic reprogramming. These modifications can directly or indirectly influence the effectiveness of immunotherapy by modulating immune checkpoints or remodeling the tumor immune microenvironment. Targeting PTMs, therefore, offers a promising avenue for improving the efficacy of ICB therapies, potentially leading to new immunotherapeutic interventions or synergistic strategies that enhance clinical outcomes for cancer patients.

The previously described studies provide a strong basis for translating basic research findings into clinical trials. In addition, a number of combinatorial strategies for cancer therapy based on the mechanisms of individual immune checkpoint PTMs have been developed and evaluated in preclinical studies [13, 14, 73, 84–86, 128, 129, 280, 281]. Continued exploration of these approaches holds promise for improving clinical outcomes and advancing the field of cancer immunotherapy.

#### PD-L1/PD-1 PTM targeted strategies

The primary objective of immune checkpoint mechanism research is to facilitate the translation of basic scientific discoveries into clinical applications. Several reagents targeting PD-L1/PD-1 PTMs have been investigated to reduce PD-L1 levels in tumors or to disrupt PD-L1 binding to PD-1. These agents include peptide mimics, GSK3β inhibitors, STM108, STM418, BMS1166, camrelizumab, mAb059c and MW11-h317Fab [13, 65, 68, 282]. In preclinical mouse tumor models, the safety and efficacy of combination therapies such as curcumin with anti-CTLA-4 antibodies [86] have also been evaluated, as curcumin promotes PD-L1 for ubiquitin degradation. Additionally, research by Bai et al. demonstrated that USP8 interacts with PD-L1 in pancreatic cancer, stabilizing PD-L1 expression by inhibiting its ubiquitin-mediated proteasomal degradation. Targeting USP8 was found to sensitize pancreatic tumors to PD-L1-targeted immunotherapy, representing a potential therapeutic strategy for pancreatic cancer treatment [103]. The combination of the α-mannosidase inhibitor swainsonine and anti-PD-L1 exerts a synergistic therapeutic effect on lung cancer and melanoma [283]. Niclosamide can enhance CTL activity by disrupting PD-1 N-linked glycosylation and significantly improve the efficacy of anti-PD-1 immunotherapy in vivo [284]. Some studies have indicated that the combined use of PKCα inhibitors and anti-PD-L1 mAb therapy may enhance the therapeutic effect in breast cancer treatment. PKCα promotes PD-L1 by phosphorylating PD-L1 S184 and reduces PD-L1 expression by activating the ubiquitin–proteasome system for ubiquitination and degradation [285].

Emerging technologies like PROTAC are revolutionizing targeted cancer therapy by leveraging the ubiquitination process to induce proteasomal degradation of specific tumor proteins [286–288]. PROTACs typically consist of a ligand of the protein of interest (POI), an E3 ubiquitin ligase ligand, and a linker, which together form a POI-PROTAC-E3 ternary complex that drives target protein degradation [289–291]. Notably, P22, a resorcinol diphenyl ether-based PROTAC molecule using pomalidomide for CRBN E3 ligase binding, degrades PD-L1 via a lysosome-dependent pathway [292]. Additionally, antibody-based PROTACs (AbTACs) recruit membrane-bound E3 ligases like RNF43 and ZNRF3 to degrade cell surface proteins such as PD-L1 [286, 293]. 21a is another PROTAC that efficiently degrades PD-L1 in a proteasome-dependent manner [294]. Innovations like lysosome-targeting chimeras (LYTACs) developed by the Bertozzi lab target extracellular and membrane proteins for lysosomal degradation, with successful application to PD-L1 [295]. GlueTAC, a covalent nanobody-based PROTAC strategy, provides a novel approach for degrading PD-L1 proteins [296].

While these technologies hold significant promise, most PROTAC-based therapies have only been validated in cellular models, and further research is needed to assess their effectiveness in in vivo tumor models and their potential for clinical application [297].

Researchers have developed STM108, a molecule that targets the glycosylation of the N192 site on PD-L1, leading to its internalization and degradation. When STM108 conjugated with MMAE, it exhibits potent antitumor effects and bystander effects on killing neighboring PD-L1-negative cancer cells, with no detectable toxicity [298]. This suggests that targeting glycosylated PD-L1 represents a promising immunotherapeutic strategy and highlights the glycosylation pathway as a potential target or biomarker for early diagnosis, though no clinical trials have yet been conducted [255]. In addition, a tumor microenvironment-activated nanoassembly that PD-L1 and CTLA-4 antagonistic aptamers are synthesized and co-assembly with glucose transporter 1 inhibitors has been demonstrated to significantly reduce PD-L1 N-linked glycosylation. In vivo, the nanoassembly can effectively inhibit N-glycosylation-driven immunosuppression and promote the response to immune checkpoint blockade therapy [299].

Beyond N-linked glycosylation, PD-L1 undergoes additional modifications, including serine/threonine phosphorylation and polyubiquitination. Horita et al. [73] also reported PD-L1 acetylation, tyrosine phosphorylation, and monoubiquitination in response to EGF stimulation. Palmitoylation/de-palmitoylation pathways offer another promising target. Advances in these researches could provide groundbreaking discoveries, improving immunotherapy outcomes and opening up new therapeutic opportunities.

#### CD47 PTM targeted strategies

The enzyme glutaminyl peptide cyclotransferase-like protein (QPCTL) has been identified as a regulator of post-translational pyroglutamate formation at the SIRPα binding site of CD47. Inhibiting QPCTL disrupts CD47 signaling, enhancing neutrophil-mediated cancer cell killing in vivo and offering a potential alternative to CD47-targeting antibodies [300]. QPCTL inhibitors, such as SEN177 and PQ912, are currently in clinical trials for neurodegenerative diseases and have shown good tolerability [301].

In summary, immune checkpoint therapy has made significant progress in clinical application, yet drug resistance remains a frequent challenge. Combination therapies can enhance immunotherapy efficacy, particularly in overcoming resistance mechanisms. Drug resistance due to PTMs of immune checkpoints is common, but targeted intervention strategies remain limited. We have outlined several mechanism studies and strategies targeting immune checkpoint PTMs, which have shown promising sensitizing effects in basic and preclinical studies. Future developments in drugs and strategies based on PTMs of immune checkpoint will be crucial for advancing clinical treatment.

## Conclusions

How PTMs affect the malignant process of tumors by regulating immune checkpoints has been a hot topic of research in recent years, and the available studies have demonstrated that PTMs of immune checkpoints play an important role in regulating tumor immune escape and affecting the efficiency of immunotherapy. Compared to transcriptional regulation, PTMs offer more flexible and dynamic control of immune checkpoint expression and function, with different types of PTMs synergizing or competing to affect immune escape mechanisms. This research has expanded our understanding of immune regulatory networks, revealed more about tumor heterogeneity, and introduced new strategies for targeting immune checkpoints in cancer therapy. However, challenges remain, particularly in determining which PTMs are critical for immune checkpoint function in clinic, especially in patients with immune therapy resistance, and suitable for drug development. While phosphorylation-related research is relatively advanced, techniques for studying other modifications, like glycosylation and palmitoylation, are still limited, requiring the development of new tools. Moreover, the transient nature of some PTMs, influenced by environmental factors, underscores the importance of accurately preserving clinical samples to reflect the in vivo modification state. Future research should aim to bridge the gap between basic PTM studies and clinical applications, focusing on multi-omics approaches to provide a comprehensive understanding of PTM roles in immune checkpoints. This could offer new insights for optimizing immunotherapy and overcoming tumor resistance mechanisms.

## Data Availability

No datasets were generated or analysed during the current study.

## Abbreviations

2DG: 2-Deoxyglucose
5-FU: 5-Fluorouracil
AbTACs: Antibody-based PROTACs
ADCs: Antibody–drug conjugates
AMPK: AMP-activated protein kinase
BTLA: B and T lymphocyte attenuator
CRLs: Cullin-RING E3 ubiquitin ligases (CRLs)
CSP: Castanospermine
CTLA-4/CD152: Cytotoxic T lymphocyte-associated protein 4
DCs: Dendritic cells
DMJ: 1-Deoxymannojirimycin
DUBs: Deubiquitinases
EGF: Epidermal growth factor
EMT: Epithelial-mesenchymal transition
ER: Endoplasmic reticulum
ERAD: ER-associated degradation
ESCC: Esophageal squamous cell carcinoma
FASN: Fatty acid synthase
FGL1: Fibrinogen-like protein 1
FKBP51s: The splice variant of FKBP5
GSK3β: Glycogen synthase kinase 3 beta
ICB: Immune checkpoint blockade
IgV: Immunoglobulin variable region
IL-6: Interleukin-6
IL-10: Interleukin-10
ITIM: Immunoreceptor tyrosine-based inhibitory motif
ITSM: Immunoreceptor tyrosine-based switch motif
JAK1: Janus kinase 1
JAMM: The JAB1/MPN/Mov34 protease family
LYTACs: Lysosome-targeting chimeras
mAbs: Monoclonal antibodies
MDSCs: Myeloid-derived suppressor cells
MIT: Mithramycin A
MJD: The Josephin domain family
NEK2: NIMA-related kinase 2
NK: Natural killer
O-GlcNAcylation: O-linked N-acetylglucosamine
OTUs: Ovarian tumor proteases
p-PROTACs: Peptide-based PROTACs
pDCs: Plasmacytoid dendritic cells
phoPS: Phosphatidylserine photoswitch element
PKC: Protein kinase C
POI: Protein of interest
PS: Phosphatidylserine
PTMs: Post-translational modifications
PVR: Poliovirus receptor
RCC: Renal cell carcinoma
SW: Swainsonine
TAMs: Tumor-associated macrophages
TGN: Trans-Golgi network
TILs: Tumor-infiltrating lymphocytes
TKIs: Tyrosine kinase inhibitors
TM: Tunicamycin
TME: Tumor microenvironment
TNBC: Triple-negative breast cancer
TSP-1: Thrombospondin-1
UCHs: Ubiquitin Carboxyl-Terminal Hydrolases
UPR: Unfolded protein response
USPs: Ubiquitin-specific peptidases
